# Improving Risk Stratification for Transient Ischaemic Attacks and Ischaemic Stroke in Patients with Coronary Artery Disease: A Combined Radiomics Analysis of Multimodal Adipose Tissue

**DOI:** 10.3390/diagnostics16010118

**Published:** 2026-01-01

**Authors:** Na Li, Shuting Wang, Hong Pan, Min Zhao, Jiali Sun, Wei Wang, Tong Zhang

**Affiliations:** 1 Department of Radiology, Fourth Affiliated Hospital of Harbin Medical University, Harbin 150001, China; linalinalinalinana@163.com (N.L.); wangshuting1751@163.com (S.W.); pan15998390057@163.com (H.P.); 2 Pharmaceutical Diagnostics, GE Healthcare, Beijing 100176, China; min.zhao@ge.com; 3 The MRI Room, First Afliated Hospital of Harbin Medical University, Harbin 150001, China; sjl71801@163.com

**Keywords:** computed tomography angiography, FAI, PFD, radiomic features, perivascular adipose tissue, IS/TIA

## Abstract

**Background/Objectives**: Patients with combined cardiovascular and cerebrovascular disease face poorer prognoses. Early, accurate assessment of the risk of cerebral ischaemic events (including transient ischaemic attacks (TIAs) and ischaemic strokes (ISs)) in patients with coronary artery disease (CAD) is therefore vital for clinical guidance. This study aims to develop a comprehensive risk assessment model for early warning in this population. **Methods**: In this study, we conducted a retrospective multicentre recruitment of CAD patients undergoing concurrent coronary CTA and cervical CTA (*n* = 326), with follow-up to observe the occurrence of cerebral ischaemic events. We performed an analysis of high-risk plaque (HRP) characteristics and subcomponent plaque in coronary and cervical arteries, measured the pericoronary fat attenuation index (FAI) and cervical perivascular fat density (PFD), and extracted corresponding radiomic features. Five models were constructed to identify the CAD patients who developed IS/TIA, respectively: Model 1—clinical characteristics; Model 2—coronary CTA parameters + Radscore_coronary_; Model 3—cervical CTA parameters + Radscore_cervical_; Model 4—Model 1 + Model 2; Model 5—Model 1 + Model 2 + Model 3. **Results**: In the cerebral ischaemia group, the prevalence of coronary and/or cervical HRP was higher than in the non-ischaemia group (28.0% vs. 26.1%, 57.0% vs. 44.0%, *p* = 0.02). Multivariate logistic regression confirmed that RCA FAI and PFD remained significant independent risk factors for IS/TIA (all *p* < 0.05). The model prediction results showed that progressively incorporating coronary and cerebral vascular risk factors into the clinical features gradually improved model performance (Model 4 vs. Model 5, AUC: 0.711 [0.645–0.777] vs. 0.821 [0.769–0.873]). Model 5 achieved a sensitivity of 0.788 [0.485–0.909] and specificity of 0.827 [0.385–0.923], demonstrating the best overall clinical benefit. **Conclusions**: RCA FAI and PFD are independent predictors of cerebral ischaemic events. By integrating clinical characteristics, coronary CTA and cervical CTA parameters, combined with Radscore_coronary_ and Radscore_cervical_, the risk stratification capability for IS/TIA in CAD patients can be significantly enhanced.

## 1. Introduction

Coronary atherosclerotic heart disease remains one of the leading causes of mortality and disability globally [1]. It is commonly attributed to myocardial ischaemia resulting from coronary atherosclerosis, often accompanied by the progression of systemic vascular disease. In recent years, numerous studies have confirmed that patients with coronary artery disease (CAD) are not only at increased risk of myocardial infarction but also face a significantly elevated risk of concomitant cerebrovascular events (e.g., transient ischaemic attack, ischaemic stroke) [2]. These conditions share fundamental pathophysiological mechanisms, including the rupture of atherosclerotic plaques, thrombosis, and systemic inflammatory responses [3,4]. This association is further intensified by the multifaceted effects of hypothermia on the cardiovascular system, particularly in colder regions [5,6]. Evidence suggests that the annual incidence of cerebral ischaemic events among patients with coronary heart disease is significantly higher in colder climates than in warmer ones, with correspondingly elevated disability and mortality rates following disease onset [7]. Patients with multisystem diseases often face more complex clinical management and experience poorer prognoses. Nonetheless, significant limitations remain in the early diagnosis and prevention of cerebral ischaemic events among patients with coronary heart disease in high-risk regions. Therefore, there is an urgent need to develop an early warning model incorporating multidimensional risk factors.

In recent years, significant progress has been made in understanding the function of perivascular adipose tissue (PVAT) [8,9], particularly in relation to cardiovascular disease [10]. Under inflammatory conditions, PVAT adipocytes detect pro-inflammatory molecules secreted by the vascular wall (e.g., IL-6, TNF-α, IFN-γ), triggering a phenotypic shift from lipid storage cells to secretory cells. This transition involves activation of lipolysis, suppression of adipogenesis [8,11], and consequently influences plaque stability [12]. Significant progress has recently been made in the study of pericoronary adipose tissue (PCAT), with multiple studies demonstrating a strong association between the inflammatory phenotype of PCAT and coronary plaque vulnerability [13,14]. Through the analysis of PCAT radiomic features (such as texture heterogeneity), local inflammatory activity can be assessed non-invasively, thereby enabling the prediction of acute coronary syndrome risk [15]. Recently, some research groups have extended their investigations to pericervical adipose tissue in order to explore its association with vulnerable plaques in the cervical arteries [16,17]. However, systematic evidence regarding whether a cross-organ association exists between the characteristics of pericoronary and pericervical adipose tissues and the occurrence of distal cerebrovascular events is lacking.

To provide new perspectives on the cross-organ interaction mechanism of “plaque–fat–cerebral ischemic events,” this study enrolled a multicentre patient cohort with suspected cardiovascular and cerebrovascular diseases. The aim is to develop imaging-based diagnostic tools and identify potential monitoring targets for individualized risk assessment and management of CAD patients residing in cold regions. Additionally, the clinical value of PVAT inflammatory status and texture characteristics in risk stratification for cerebral ischemic events will be evaluated.

## 2. Materials and Methods

### 2.1. Study Population

This study employed a multicentre retrospective analysis method, incorporating clinical case data from two medical institutions (with four independent campuses located in different geographical locations) in Northern China. The study population consisted of patients who concurrently underwent both coronary computed tomography angiography (CTA) and cervical CTA imaging examinations between June 2020 and December 2023 due to suspected cardiovascular and cerebrovascular diseases (with an interval of ≤3 months between examinations) and were diagnosed with coronary atherosclerotic heart disease by coronary CTA. The exclusion criteria were as follows: (1) patients with a history of coronary artery bypass grafting or stenting; (2) patients without coronary atherosclerotic heart disease as confirmed by coronary CTA and patients with atrial fibrillation; (3) those who had undergone carotid endarterectomy, carotid stenting, or implantation of artificial blood vessels; (4) patients with previous old cerebral infarction and cerebral haemorrhage; and (5) incomplete clinical or follow-up data, missing images, or images of insufficient quality for parameter measurement.

### 2.2. Assessment of Cerebral Ischaemic Events

**Figure 1:**
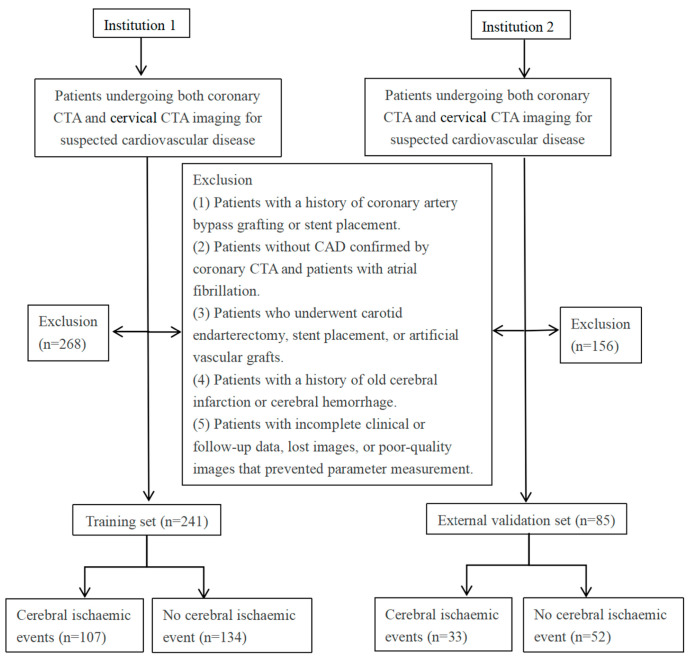
Flowchart illustrating the inclusion and exclusion criteria of the study population. CTA: computed tomography angiography; CAD: coronary artery disease.

The primary outcome of this study was first-ever cerebral ischaemic events, defined as ischaemic stroke (IS) or transient ischaemic attack (TIA). The observation period spanned from the time of cervical CTA examination until January 2025, including first-ever cerebral ischaemic events that occurred contemporaneously (i.e., during the same hospitalisation) as the cervical CTA as well as those that emerged during follow-up. Diagnostic criteria were based on the 2021 Journal of the American Medical Association guidelines for diagnosis and management [18]. We adopted a multimodal observation and follow-up strategy: (1) Imaging confirmation—neuroimaging evidence was obtained through head CT or MRI; assessment was performed according to guideline standards to evaluate for the presence of infarcts or causative vascular lesions, thereby confirming the diagnosis of ischaemic stroke. (2) Structured telephone follow-up. (3) Medical record review: retrieve electronic medical records from the medical information platform and query outpatient/inpatient records. The flowchart of the study design is shown in Figure 1. A total of 326 patients with CAD were included. Of these, 241 patients from Institution 1 comprised the training set, while 85 patients from Institution 2 formed the validation set. The study was approved by the local Institutional Review Board (No. 2025-LLSC-45), and the requirement for informed consent was waived due to its retrospective design.

### 2.3. Clinical Data Evaluation

Baseline characteristics (including age, sex, body mass index (BMI), hypertension, diabetes mellitus, hyperlipidaemia [19], smoking and alcohol consumption history, and heart rate) were extracted from hospitalisation records. Laboratory results for blood glucose, total cholesterol, triglyceride levels, and triglyceride-glucose index (TyG) [20] were also collected. Definitions of the relevant clinical indicators are provided in the Supplementary Materials.

### 2.4. CTA Inspection Methods

At Institution 1, coronary CTA was performed using a Siemens SOMATOM Force CT (Siemens Healthcare Ltd., Forchheim, Germany), an Aquilion ONE 320-row CT (Canon Medical, Otawara, Japan), and a Neusoft NeuViz Epoch CT scanner (Neusoft Medical Systems Co., Ltd., Shenyang, China). At Institution 2, imaging was conducted using a Philips Brilliance iCT 256-slice (Philips Healthcare, Amsterdam, The Netherlands). Cervical CTA was performed using the same scanners described above. Detailed scanning parameters are provided in the Supplementary Materials. To minimize technical variations between different scanners, we performed standardized preprocessing on all images. First, linear interpolation techniques were used to resample the images, unifying the voxel size to an isotropic 1.0 × 1.0 × 1.0 mm^3^. Second, a Gaussian blur image filter was applied to smooth the images, effectively suppressing noise.

### 2.5. Coronary CTA Data Analysis

Based on the coronary CTA imaging data, plaque subcomponent volumes in the three major coronary arteries (including LM + LAD, LCX, and RCA) were measured using CardioDoc^®^ software (version 8.09.1229, Beijing Shukun Technology Co., Ltd., Beijing, China). Quantified parameters included total plaque volume, calcified plaque volume (>350 HU), and non-calcified plaque volume, with the latter comprising fibrous plaque (131–350 HU), fibro-lipid plaque (31–130 HU), and lipid core (≤30 HU). Corresponding coronary CTA parameter measurements are presented in Figure S1.

Plaque morphological characteristics at the site of greatest stenosis were analysed on a lesion-level basis for each patient. Included minimum lumen area (MLA), degree of stenosis, and high-risk plaque (HRP) features. A plaque was classified as high-risk if two or more of the following features were present: lipid core (low-density plaque), napkin-ring sign, spotty calcification, and positive remodelling.

### 2.6. Cervical CTA Evaluation

**Figure 2:**
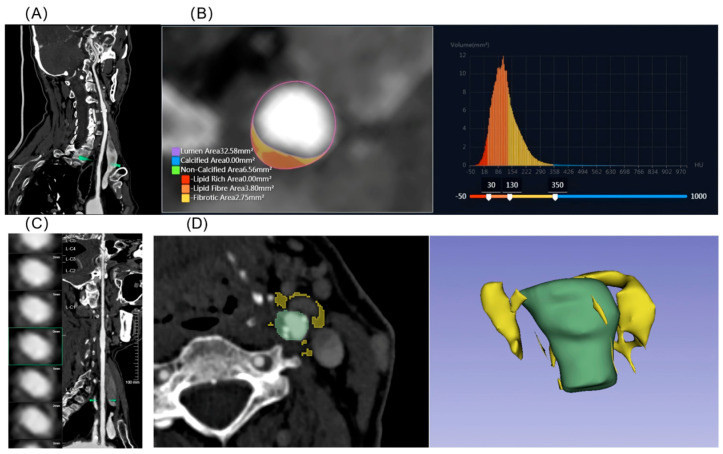
Illustrative examples of cervical artery CTA analysis. (**A**) Curved planar reformation of the carotid artery, (**B**) Plaque composition analysis with red indicating lipid-rich plaque, yellow representing fibro-fatty plaque, orange denoting fibrous plaque, and blue corresponding to calcified plaque. (**C**) Analysis of perivascular adipose tissue at the site of maximal stenosis identified on the curved reformation. (**D**) Cross-sectional view of PFD measurement and 3D ROI volume rendering, in which the yellow colour represents the pericervical adipose tissue. These cervical artery parameter measurements are from the same patient shown in Figure S1, who subsequently developed an ischaemic stroke 23 months later. PFD: pericarotid fat density.

Quantitative analysis of cervical artery plaque was assessed using the uAI Discover Cerebral CTA (version R001, United Imaging Intelligent Healthcare, Shanghai, China) (Figure 2), including bilateral common carotid artery (CCAs), internal carotid artery (ICAs) and vertebral artery (VAs). Total plaque volume, calcified plaque volume, and non-calcified plaque volume (including fibrous plaque, fibro-lipid plaque, and lipid core) were measured using the same quantitative criteria applied to coronary CTA plaques. Stenosis quantification was performed on axial images reconstructed from curved planar reformation images.

Lesion analysis was conducted at the site of the narrowest lumen, with calculation of the degree of stenosis, MLA. Cervical artery plaques were classified as high-risk based on morphological characteristics. High-risk features included soft plaques, plaque ulceration, neovascularization (plaque enhancement), and plaque thickness ≥3 mm (Supplementary Materials) [21]. All imaging parameters were independently evaluated by two radiologists, each with over five years of experience in vascular imaging. Both radiologists were blinded to the patients’ clinical information. In cases of disagreement, a senior radiologist with more than ten years of experience in vascular imaging was consulted to reach a consensus.

### 2.7. PVAT Analysis

The Fat Attenuation Index (FAI) is derived by adjusting technical parameters to calculate the average attenuation of PCAT. We automatically determined the region of interests (ROI) and FAI of the three major coronary arteries using dedicated analysis software (uAI Research Portal, version 20250130, United Imaging Intelligent, Shanghai, China). The measurement range for LAD FAI and LCX FAI was the proximal 40 mm, while that for RCA FAI spanned from 10 mm to 50 mm from the ostium. The radial distance was defined as the vessel diameter, encompassing all voxels within the CT attenuation range of −190 HU to −30 HU (Figure S1).

Pericervical adipose tissue was analysed using 3D Slicer (v 5.6.2), with the vessel wall boundary tracked and delineated layer by layer across multiple consecutive slices. The ROI for pericervical adipose tissue was manually delineated and defined as adipose tissue located within a radial distance (equal to the average diameter of the target vessel) from the outer vessel wall. The assessment was cantered at the site of the most severe stenosis, covering a length of the cervical artery segment with atherosclerotic plaque. All voxels within the CT attenuation range of −190 HU to −30 HU were included. The mean CT attenuation value within the ROI was defined as the cervical perivascular fat density (PFD). In the absence of cervical artery stenosis, the PFD was measured bilaterally with the carotid bifurcation as the centre, and the average value was calculated [22].

### 2.8. Radiomics Feature Extraction

The images were normalised and resampled using a preprocessor, and grey-level quantisation was applied for radiomic phenotyping analysis. Previous studies have demonstrated that RCA PCAT can serve as a surrogate for whole-heart adipose tissue [23]. Accordingly, the U-Net deep learning algorithm implemented on the Research Portal V1.6 platform (United Imaging Intelligence, Shanghai, China) was used to automatically segment and mask RCA PCAT images. Radiomic analysis of the pericervical adipose tissue region was conducted in Jupyter Notebook (Version 7.4.4), with feature extraction performed using the open-source PyRadiomics library (Version 3.0.1). In total, 2264 and 1595 radiomic features were extracted from the two respective regions, and all features were standardised using Z-scores. The detailed procedures for feature extraction and selection are provided in the Supplementary Materials.

### 2.9. Statistical Analyses

Principal component analysis (PCA) was applied for dimensionality reduction and visualisation to assess clustering of samples by institution and to reveal the intrinsic data structure. Correlation analyses were performed using Spearman’s test, univariate and multivariable logistic regression were used to evaluate associations between clinical and CTA characteristics and outcome events. Radiomics features were selected using LASSO regression. Other statistical methods are detailed in the Supplementary Materials. All statistical analyses were conducted using R software (version 4.5.0), and two-sided *p*-values < 0.05 were considered statistically significant.

## 3. Results

### 3.1. Baseline Characteristics of Patients

During a median observation and follow-up period of 22 [15.00–31.00] months, a total of 326 patients with CAD and complete clinical and imaging data were included in this study. Among them, 107 (44.4%) cases in the training set (*n* = 241) experienced cerebral ischaemic events during observation and follow-up, and 33 (38.8%) cases in the validation set (*n* = 85) experienced cerebral ischaemic events. The mean age of all patients was 63.30 (9.17) years.

Table 1, Tables S1 and S2 present the baseline clinical characteristics of patients with CAD, stratified by the presence or absence of cerebral ischaemic events across the two institutional cohorts. There were no significant differences in baseline characteristics between the training and validation sets (*p* > 0.05) (Table S1). However, within the training set, the prevalence of hypertension was significantly higher among patients who experienced cerebral ischaemic events (*p* = 0.045).

### 3.2. Characterisation of Coronary and Cervical CTA Parameters

The PCA revealed no significant separation between samples from the two institutions along the first two principal components (PC1 and PC2) (Figures S2 and S3). The data points were well-mixed in the PCA plots, indicating minimal inter-institutional technical variation and supporting the feasibility of combining the datasets for analysis.

In the training set (Table 2), patients who experienced a cerebral ischaemic event had a significantly greater volume of non-calcified plaque in the LM + LAD segment compared to those without an event (32.6 [11.8; 62.9] vs. 19.6 [3.51; 54.5], *p* = 0.025), with the difference primarily attributable to lipid (*p* = 0.007) and fibro-lipid plaque volumes (*p* = 0.013). FAI values of the LAD, LCX, and RCA were higher in the ischaemic event group; however, a statistically significant difference was observed only in the RCA FAI measurements [−79.70 (10.6) vs. −83.34 (10.3), *p* = 0.008].

Analysis of cervical artery CTA parameters revealed that significant differences in calcified and non-calcified plaque volumes between the cerebral ischaemic and non-ischaemic groups were observed only in the right CCA (*p* = 0.007 and *p* = 0.048, respectively) (Table 3). The difference in non-calcified plaque volume was primarily attributable to the fibrous plaque component (*p* = 0.016). Pericervical adipose tissue demonstrated higher PFD values in the cerebral ischaemic event group compared to the non-ischaemic group (−68.11 [−74.33; −61.13] vs. −70.77 [−79.38; −64.94], *p* = 0.017). CTA characteristics for the validation group are presented in Table S3.

**Figure 3:**
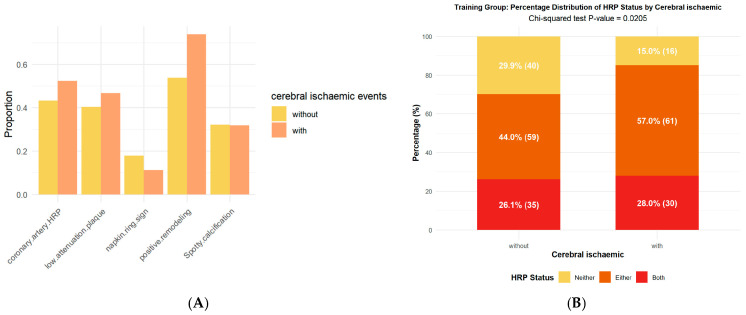
Analysis of coronary HRP and cervical arterial HRP. (**A**) Probability plot of the occurrence of coronary HRP features with and without the cerebral ischaemic event occurring with significant differences only in the feature of positive remodelling. (**B**) Probability plot of HRP occurrence in the group with (right) and without (left) cerebral ischaemic events, with yellow for, i.e., neither coronary nor cervical arterial HRP, orange for presence of coronary HRP or presence of cervical arterial HRP, and red for presence of both coronary and cervical arterial HRP. HRP: high-risk plaque.

In the analysis of HRP in the coronary and cervical arteries, the presence of a cerebral ischaemic event was significantly associated only with the feature of positive remodelling (Table S5, Figure 3). However, the likelihood of exhibiting both coronary and cervical artery HRP (28.0% vs. 26.1%), as well as that of having either (57.0% vs. 44.0%), was higher in the cerebral ischaemic event group compared to the non-event group (*p* = 0.02).

Twenty patients were randomly selected to evaluate intra- and inter-observer reliability for plaque characteristics and PFD measurements. The results demonstrated Kappa values greater than 0.85 for all high-risk plaque features, while intra- and inter-observer intraclass correlation coefficients (ICCs) for PFD exceeded 0.90, indicating excellent measurement reliability (Figure S4).

### 3.3. Plaque Characteristics and Perivascular Adipose Tissue Correlation Analysis

**Figure 4:**
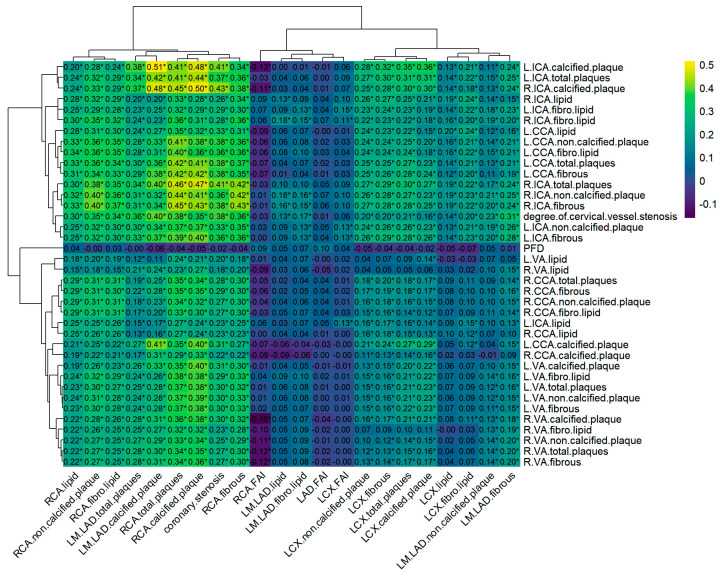
Heatmap of coronary plaque characteristics and FAI correlation with cervical artery plaque characteristics and PFD. The horizontal coordinates shown in the figure are the coronary CTA measurements and the vertical coordinates are the cervical artery CTA measurements. *: *p* < 0.05. The numerical values in the figure represent Spearman correlation coefficients. LM: Left Main, LAD: left anterior descending artery, LCX: left circumflex artery, RCA: right coronary artery, FAI: fat attenuation index, R: right, L: left, CCA: common carotid artery, ICA: internal carotid artery, VA: vertebral artery, PFD: perivascular fat density.

To investigate the associations between coronary and cervical atherosclerotic plaques, we conducted a Spearman correlation heatmap of CTA parameters (Figure 4). A general correlation was observed between various components of RCA plaques and cervical arterial plaques (*p* < 0.05), while correlations involving LM + LAD were mainly limited to calcified plaque and total plaque volumes. Notably, calcified plaques in the RCA and LM + LAD showed stronger correlations with the presence of calcified plaques in the bilateral ICAs (RCA and L-ICA: r = 0.48; RCA and R-ICA: r = 0.50; LM + LAD and L-ICA: r = 0.51; LM + LAD and R-ICA: r = 0.48; all *p* < 0.05). No significant correlation was observed between coronary FAI and cervical PFD (*p* > 0.05).

### 3.4. Risk Analysis of Cerebral Ischaemic Events

**Figure 5:**
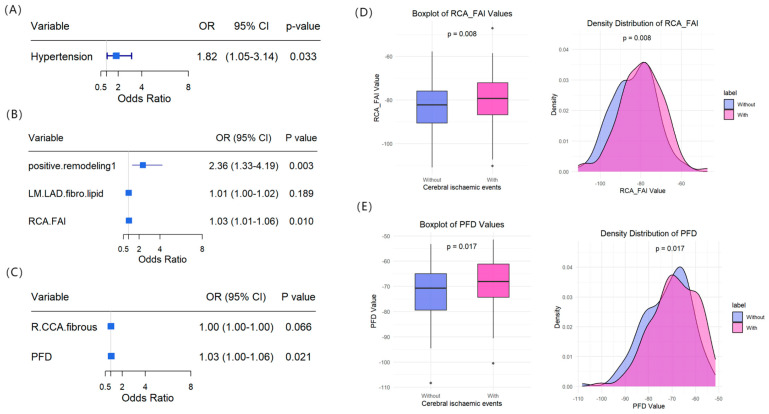
Risk factor analysis for the occurrence of cerebral ischaemic events. (**A**–**C**) demonstrate forest plots of multiple logistic regression analysis based on clinical characteristics, coronary CTA parameters, and cervical CTA parameters, respectively (note: 1 indicates the presence of positive remodeling in (**B**)), and (**D**,**E**) show the distribution characteristics of RCA FAI and PFD in the cerebral ischemic event versus non-event groups, respectively: the left box-and-line plot demonstrates the differences between groups, and the right densitometric plot presents the distribution of the measured parameters. The distribution of the remaining measured parameters can be seen in Supplementary Materials. CTA: computed tomography angiography, RCA: right coronary artery, FAI: fat attenuation index, PFD: perivascular fat density.

Clinical characteristics, coronary CTA parameters, and cervical artery CTA parameters were included in both univariate and multivariate logistic regression analyses. Univariate analysis revealed that cerebral ischaemic events were significantly associated with hypertension (OR = 1.820), LM + LAD fibro-lipid plaque volume (OR = 1.012), positive remodelling (OR = 2.430), RCA FAI (OR = 1.034), right CCA fibrous plaque volume (OR = 1.002), and PFD (OR = 1.002) (all *p* < 0.05; Tables S6–S8). In multivariate regression analysis, hypertension, positive remodelling, RCA FAI, and PFD remained statistically significant as independent risk factors for the occurrence of cerebral ischaemic events (all *p* < 0.05; Figure 5A–C). These results indicate that elevated RCA FAI and cervical artery PFD are independent predictors of cerebral ischaemic events (Figure 5D,E and Figure S5).

### 3.5. Radiomic Feature Analysis of Perivascular Adipose Tissue

Through logistic regression (retaining features with *p* < 0.1) and the elimination of highly correlated features, 625 pericoronary and 400 pericervical adipose tissue radiomic features were retained. Subsequent LASSO regression identified 8 coronary and 16 cervical perivascular adipose tissue radiomic features (Figures S6 and S7), which were used to calculate Radscore_coronary_ and Radscore_cervical_ based on the non-zero coefficients (Figure S8). Details of the radiomic feature selection process and the calculation formulae are provided in the Supplementary Materials. Furthermore, multivariate logistic regression analysis further confirmed that both Radscore_coronary_ and Radscore_cervical_ were independent risk factors for cerebral ischaemic events (OR = 10.776 and OR = 12.656, respectively; both *p* < 0.001) (Figure S9).

### 3.6. Assessment of Models for Cerebral Ischaemic Events Using Multimodal Clinical Imaging Lipid Radiomics Metrics

**Figure 6:**
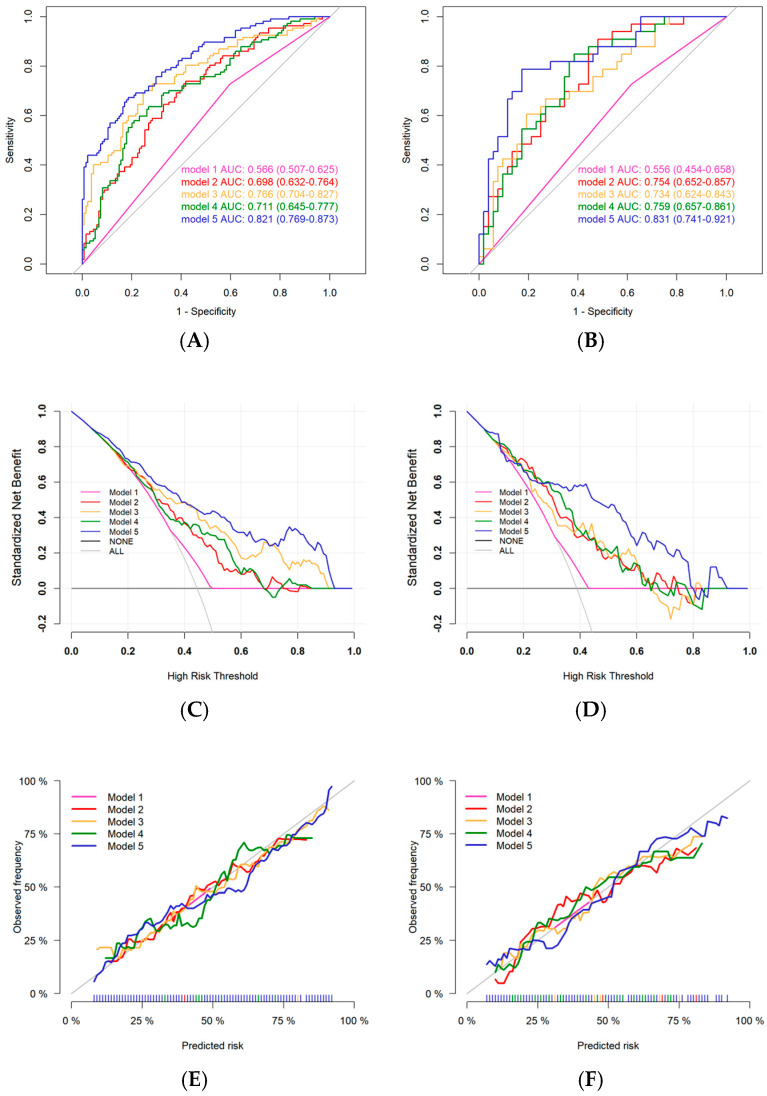
Comparison of Model Performance for Assessing Cerebral Ischaemic Events in Patients with CAD. The illustration includes: the ROC curves (**A**,**B**), decision curve analysis (**C**,**D**), and calibration curves (**E**,**F**). Where ACE is the training set and BDF is the validation set. Where model 1 is based on clinical features, model 2 is based on coronary CTA features + Radscore_coronary_, model 3 is based on cervical arterial CTA features + Radscore_cervical_, model 4 is model 1 + model 2, and model 5 is model 1 + model 2 + model 3, that is, a comprehensive model that includes all features. CAD: coronary artery disease, CTA: computed tomography angiography, IS: ischaemic strokes, TIA: transient ischaemic attacks, RCA: right coronary artery, FAI: fat attenuation index, PFD: perivascular fat density.

To evaluate the risk of IS or TIA in patients with CAD, we developed risk assessment models using parameters with *p* < 0.05 identified in univariate logistic regression analysis, along with the radiomics scores (Radscore_coronary_ and Radscore_cervical_). Five models were constructed for comparison: Model 1—based on clinical features alone; Model 2—combining coronary CTA features with Radscore_coronary_; Model 3—combining cervical CTA features with Radscore_cervical_; Model 4—an integrated model combining Model 1 and Model 2; Model 5—a comprehensive multimodal model (i.e., Model 1 + Model 2 + Model 3). Logistic regression was used to construct the models. The results of ROC curves showed (Figure 6) that Model 3 [AUC: 0.766 (0.704–0.827)] outperformed the diagnostic performance of Model 2 [AUC: 0.698 (0.632–0.764)] in the training set, whereas both had similar diagnostic efficacy in the validation set [Model 3 vs. Model 2 AUC: 0.734 (0.624–0.843) vs. 0.754 (0.652–0.857)]. Model performance improved progressively with the sequential incorporation of coronary and cervical artery risk features into the clinical baseline model [Model 4 vs. Model 5 training set AUC: 0.711 (0.645–0.777) vs. 0.821 (0.769–0.873)]. Decision curve analysis (DCA) indicated that the comprehensive Model 5 provided the greatest clinical net benefit across a range of threshold probabilities. Additionally, calibration curve analysis confirmed that all models demonstrated good agreement between predicted and observed outcomes, suggesting excellent model fit (Figure 6).

Table 4 compares the performance of the different models on the validation set. Model 5 achieved the highest overall diagnostic performance, with a sensitivity of 0.788 (0.485–0.909), specificity of 0.827 (0.385–0.923), positive predictive value of 0.743 (0.577–0.889), and negative predictive value of 0.860 (0.742–0.944). The corresponding results for the training set are presented in Table S9.

In the analysis of the diagnostic efficacy of perivascular adipose tissue for cerebral ischaemic events (Figure S10), Radscore_cervical_ demonstrated superior predictive performance [validation group AUC: 0.726 (0.615–0.837)] compared to Radscore_coronary_ [AUC: 0.713 (0.599–0.826)]. Both scores outperformed the RCA FAI and PFD. In the incremental value analysis of PVAT radiomics (Figure S11), a significant upward trend in the model’s AUC was observed as Radscore_coronary_ and Radscore_cervical_ were sequentially added to the clinical and CTA parameters.

## 4. Discussion

Building on previous research into the association between PVAT and cardiovascular and cerebrovascular diseases, this study further investigated the risk assessment value of PCAT and PFD for cerebral ischaemic events. Previous studies have demonstrated the prognostic value of PCAT in predicting acute coronary syndromes and all-cause mortality [23,24]. Some studies have also reported the association between PFD measured by the cross-sectional area method and cerebrovascular events [25]. However, studies evaluating both PVAT sites simultaneously remain limited. Given the high prevalence of CAD and cerebral ischaemic events in populations residing in cold regions, this study integrated PVAT indices from both anatomical sites and further analysed their radiomic texture features to facilitate the early identification of CAD patients at risk of IS/TIA. The results demonstrated that both RCA FAI and PFD were independent risk factors for IS/TIA. Moreover, the combined model incorporating both Radscore_coronary_ and Radscore_cervical_ achieved the highest predictive performance (AUC = 0.821), offering a novel combination of imaging biomarkers for early risk stratification of cardiovascular and cerebrovascular events.

Patients who experienced cerebral ischaemic events exhibited higher levels of chronic inflammation in the coronary vascular system. Among the inflammatory indicators assessed, only the FAI of the RCA was identified as a significant risk factor for these events. Furthermore, as previous studies have confirmed that RCA FAI reliably reflects systemic cardiovascular inflammation [23], the RCA ROI was selected as the target area for radiomic feature extraction in this study. The measurement of PFD in this study followed the protocol described by Lan, Yu and Zhang et al. [17,22]. Zhang et al. demonstrated a significant association between elevated PFD and the presence of cerebrovascular symptoms, suggesting its potential as an imaging biomarker for symptomatic carotid plaque [17]. In our study, patients who experienced cerebral ischaemic events exhibited higher PFD values, further supporting the diagnostic value of PFD in identifying IS/TIA.

In coronary and cervical CTA plaque characterisation, the volume of non-calcified plaque in the LM + LAD was greater in patients who experienced cerebral ischaemic events. This finding is likely attributable to the higher prevalence of plaque in the proximal segment of the LAD, which may be influenced by its anatomical position and haemodynamic properties. The formation of plaques in the proximal segments may be closely associated with alterations in local shear stress and endothelial dysfunction. Furthermore, non-calcified plaques are typically considered vulnerable and carry a higher risk of rupture, which may account for their association with more severe clinical outcomes [26,27]. Our findings revealed that patients in the cerebral ischaemic event group had significantly greater volumes of both calcified and non-calcified plaques in the right CCA. This observation may be explained by the anatomical origin of the right CCA from the brachiocephalic trunk, which shares a common origin with the subclavian artery supplying the right upper limb. Because the majority of individuals are right-handed, frequent right upper limb activity can induce turbulent blood flow within the brachiocephalic trunk. Such persistent hemodynamic disturbance may contribute to endothelial injury in the right CCA, thereby promoting atherosclerotic plaque formation. Furthermore, non-calcified plaques may partially reflect fibrous tissue encapsulating intraplaque haemorrhage (IPH) and lipid-rich necrotic cores (LRNC). In patients with cerebral ischaemic events, plaques may concurrently exhibit both LRNC and IPH, two pathological features that are strongly associated with an increased risk of stroke [28,29]. However, due to partial volume effects in CT imaging, the density of fibrous tissue is averaged with surrounding components, often resulting in its misclassification within the fibrous plaque range [30,31].

Research has established a close interrelationship between cardiovascular and cerebrovascular diseases, which frequently share common risk factors, including atherosclerosis, thrombosis, and systemic inflammation [32]. We found that the proportion of patients with HRPs in both coronary and cervical arteries (or in at least one of these sites) was significantly higher among those who experienced cerebral ischaemic events. These findings are important for guiding the clinical recognition of high-risk vascular plaque features. In recent years, with the expanding application of radiomics and artificial intelligence in cardiovascular and cerebrovascular risk assessment, numerous studies have focused on extracting textural and structural information from routine imaging modalities such as CTA. For example, Zhao and Ji-Yan et al. successfully differentiated symptomatic from asymptomatic cerebral plaques using a radiomics model based on PVAT [33,34]. Similarly, Le et al. compared radiomics and deep learning approaches with conventional calcium scoring and demonstrated potential advantages in the detection of cervical artery disease [35]. In the present study, the stepwise incorporation of radiomic features derived from PVAT in both the coronary and cervical arteries further improved the stratification of cerebral ischaemic event risk.

Radscore_coronary_ primarily integrates density distribution and textural features of PCAT under various image filters, such as Laplacian sharpening and discrete Gaussian filtering. Among these, features from the Laplacian sharpening first-order series contribute positively, with higher values indicating the potential presence of more focal high-density areas within PCAT, which may reflect inflammatory cell infiltration, microcalcification, fibrosis or other unhealthy conditions. Radscor_ecervical_ comprehensively reflects morphological, wavelet-based multi-scale texture features, and grey-level distribution information of pericervical adipose tissue. The significant positive contribution of original_shape_Sphericity suggests that a more spherical shape of fat deposition is associated with higher risk, potentially indicating a focal and cluster-like abnormal fat distribution pattern. Meanwhile, wavelet-HHL_firstorder Skewness, the strongest positive contributor, indicates that an increase in positive skewness may correspond to focal abnormal high-signal areas, possibly related to local oedema, haemorrhage or severe inflammation. Both scores non-invasively reveal the level of inflammation and atherosclerotic activity in the patient’s arterial system, thereby enabling effective monitoring of the risk of cerebral ischemic events.

### Limitations

This study has several limitations. First, although data were collected from two medical centres, the sample size and geographical coverage were limited and may not fully reflect the characteristics of the broader population. Whilst the model demonstrated good performance in both internal and external validation, the generalisability of the findings may remain constrained by the moderate size of the validation cohorts. Future studies should incorporate larger, multicentre populations to further validate these findings. Second, the follow-up and observation period in this study was relatively short, which may constrain the comprehensive assessment of the long-term predictive performance of the indicators. Furthermore, during the retrospective data collection phase, complete and reliable timing information for the occurrence of events was not available. The absence of follow-up time data may hinder a full reflection of the dynamic risk of event occurrence. Future studies should incorporate longer and more precise time-followed observations to further validate the stability and assessment accuracy of the model. Third, due to the absence of a reliable automated sketching tool in this study, the ROI of PFD was delineated manually and semi-automatically, which may introduce subjective bias and measurement error. Future research could incorporate reliable AI-assisted segmentation techniques to enhance the accuracy and reproducibility of ROI delineation. Fourth, due to the retrospective and multi-centre design of this study, systematic control and assessment of the confounding factor of patients’ medication use (e.g., lipid-lowering drugs) was not feasible, which may have introduced a degree of confounding bias into the results. In future research, a prospective design will be adopted to collect more comprehensive covariate data, thereby further enhancing the accuracy of the study. Fifth, this study only included participants residing in cold regions, a population inherently at higher risk for cardiovascular disease, meaning the findings may not be generalisable to populations in other climates. Furthermore, all enrolled participants underwent both coronary and cervical CTA examinations due to suspected cardiovascular disease. This design resulted in a cohort with a relatively high incidence of cerebral ischemic events, potentially introducing selection bias. It should be emphasized that in clinical practice, patients indicated for both CTA examinations typically constitute a high-risk subgroup for cardiovascular and cerebrovascular diseases. Therefore, the radiomics features derived from these CTA images could yield more targeted imaging evidence for disease assessment and risk stratification within this specific high-risk population.

## 5. Conclusions

Based on assessments from coronary and cervical CTA, RCA FAI and PFD may serve as independent risk indicators for cerebral ischaemic events in patients with coronary artery disease residing in cold climates. By integrating imaging parameters from both coronary and cervical CTA, along with Radscore_coronary_ and Radscore_cervical_, the risk stratification for IS/TIA in CAD patients can be significantly enhanced.

## Figures and Tables

**Table 1:** Clinical baseline characteristics of patients with or without cerebral ischemic events (training and validation sets).

Patients	Training Set (*n =* 241)			Validation Set (*n =* 85)		
	Cerebral Ischaemic Events (*n =* 107)	No Cerebral Ischaemic Events (*n =* 134)	*p*	Cerebral Ischaemic Events (*n =* 33)	No Cerebral Ischaemic Events (*n =* 52)	*p*
Age (years)	63.10 (9.14)	63.50 (9.73)	0.695	63.00 (7.88)	63.50 (8.72)	0.773
Male	72 (67.3%)	77 (57.5%)	0.497	23 (69.7%)	31 (59.6%)	0.478
BMI (kg/m^2^)	25.00 (3.40)	24.80 (3.18)	0.683	25.00 (3.51)	24.50 (3.11)	0.517
Hypertension	78 (72.9%)	80 (59.7%)	0.045	24 (72.7%)	32 (61.5%)	0.409
Diabetes mellitus	41 (38.3%)	41 (30.6%)	0.263	15 (45.5%)	17 (32.7%)	0.340
Smoking	26 (24.3%)	38 (28.4%)	0.574	11 (33.3%)	14 (26.9%)	0.698
Hyperlipidaemia	65 (60.7%)	74 (55.2%)	0.465	17 (51.5%)	30 (57.7%)	0.738
Heart rate (times/minute)	80.00 [70.00;86.00]	78.00 [68.20;85.00]	0.221	74.70 (12.40)	77.40 (10.90)	0.296
Blood glucose (mmol/L)	6.55 (2.12)	6.20 (2.15)	0.208	6.33 (1.38)	6.50 (2.54)	0.691
Total cholesterol (mmol/L)	4.60 (1.23)	4.77 (1.24)	0.288	4.35 (1.03)	4.65 (1.29)	0.226
Triglycerides (mmol/L)	1.42 [1.08;1.91]	1.43 [1.11;2.03]	0.637	1.62 [0.98;1.94]	1.38 [0.99;2.01]	0.878
TyG Index	8.87 [8.47;9.18]	8.81 [8.48;9.18]	0.687	8.87 (0.63)	8.85 (0.61)	0.837
Pharmacotherapy (Hypertension)	52 (48.6%)	55 (41.0%)	0.297	17 (51.5%)	20 (38.5%)	0.338

BMI = body mass index.

**Table 2:** Characterisation of imaging parameters for coronary CTA (training set).

	No Cerebral Ischaemic Events (*n =* 134)	Cerebral Ischaemic Events (*n =* 107)	*p*
LM + LAD total plaques	52.6 [11.6;122]	71.8 [25.5;146]	0.107
LM + LAD calcified plaque	14.6 [0.29;75.1]	17.0 [1.33;76.2]	0.561
LM + LAD non-calcified plaque	19.6 [3.51;54.5]	32.6 [11.8;62.9]	0.025
LM + LAD lipid	0.92 [0.00;5.77]	2.48 [0.32;8.04]	0.007
LM + LAD fibrous	9.16 [2.78;22.5]	13.5 [4.69;28.1]	0.092
LM + LAD fibro-lipid	6.08 [0.20;21.4]	12.7 [1.34;27.1]	0.013
LCX total plaques	0.00 [0.00;24.8]	0.00 [0.00;21.4]	0.609
LCX calcified plaque	0.00 [0.00;10.3]	0.00 [0.00;12.3]	0.506
LCX non-calcified plaque	0.00 [0.00;8.14]	0.00 [0.00;9.78]	0.708
LCX lipid	0.00 [0.00;0.09]	0.00 [0.00;0.00]	0.762
LCX fibrous	0.00 [0.00;5.00]	0.00 [0.00;4.75]	0.677
LCX fibro-lipid	0.00 [0.00;0.88]	0.00 [0.00;0.72]	0.240
RCA total plaques	23.7 [0.00;156]	38.5 [0.00;151]	0.493
RCA calcified plaque	5.06 [0.00;58.9]	8.12 [0.00;60.1]	0.711
RCA non-calcified plaque	10.4 [0.00;67.0]	16.1 [0.00;85.1]	0.471
RCA lipid	0.40 [0.00;6.56]	0.30 [0.00;8.98]	0.738
RCA fibrous	5.86 [0.00;37.3]	6.68 [0.00;33.7]	0.447
RCA fibro-lipid	3.39 [0.00;20.9]	3.59 [0.00;35.1]	0.511
LAD FAI (HU)	−80.73 (9.10)	−78.80 (8.11)	0.084
LCX FAI (HU)	−75.98 (9.05)	−73.85 (10.0)	0.088
RCA FAI (HU)	−83.34 (10.3)	−79.70 (10.6)	0.008

The volume units for all plaques in the table are in mm^3^. LM: Left Main, LAD: left anterior descending artery, LCX: left circumflex artery, RCA: right coronary artery, FAI: fat attenuation index.

**Table 3:** Characterisation of imaging parameters for cervical artery CTA (training set).

	No Cerebral Ischaemic Events (*n =* 134)	Cerebral Ischaemic Events (*n =* 107)	*p*
L.CCA calcified plaque	3.00 [0.00;53.3]	6.69 [0.00;58.4]	0.411
L.CCA non-calcified plaque	105 [0.00;349]	95.4 [0.00;396]	0.590
L.CCA fibrous	36.2 [0.00;112]	43.5 [0.00;125]	0.504
L.CCA fibro-lipid	42.6 [0.00;163]	32.7 [0.00;193]	0.604
L.CCA lipid	1.48 [0.00;25.3]	0.46 [0.00;23.7]	0.680
L.CCA total plaques	129 [0.00;403]	143 [0.00;457]	0.521
L.ICA calcified plaque	22.3 [0.37;110]	44.0 [2.08;158]	0.153
L.ICA non-calcified plaque	10.2 [0.40;76.4]	19.2 [4.03;157]	0.069
L.ICA fibrous	10.1 [0.38;43.1]	16.8 [4.03;98.2]	0.064
L.ICA fibro-lipid	0.06 [0.00;24.0]	0.54 [0.00;47.0]	0.174
L.ICA total plaques	65.7 [4.11;312]	92.5 [8.43;439]	0.167
R.CCA calcified plaque	0.00 [0.00;14.8]	3.72 [0.00;86.6]	0.007
R.CCA non-calcified plaque	4.65 [0.00;147]	60.8 [0.00;189]	0.048
R.CCA fibrous	3.20 [0.00;54.1]	33.0 [0.00;78.8]	0.016
R.CCA fibro-lipid	0.20 [0.00;77.4]	13.8 [0.00;104]	0.054
R.CCA lipid	0.00 [0.00;4.97]	0.01 [0.00;9.66]	0.168
R.CCA total plaques	8.12 [0.00;185]	104 [0.00;254]	0.035
R.ICA calcified plaque	30.2 [0.00;124]	40.0 [2.70;203]	0.079
R.ICA non-calcified plaque	8.98 [0.00;110]	17.6 [3.28;200]	0.068
R.ICA fibrous	8.34 [0.00;72.8]	16.8 [3.28;85.8]	0.053
R.ICA fibro lipid	0.00 [0.00;32.9]	0.29 [0.00;72.4]	0.073
R.ICA total plaques	70.5 [0.00;325]	106 [13.4;350]	0.124
PFD (HU)	−70.77 [−79.38;−64.94]	−68.11 [−74.33;−61.13]	0.017

All plaque volumes in the table are in mm^3^. More cervical plaque measurement data are provided in the Supplementary Materials Table S4. R: right, L: left, CCA: common carotid artery, ICA: internal carotid artery, PFD: perivascular fat density.

**Table 4:** Diagnostic performance metrics for different models (validation set).

Model	AUC (95% CI)	SEN (95% CI)	SPE (95% CI)	PLR (95% CI)	NLR (95% CI)	PPV (95% CI)	NPV (95% CI)
Model 1	0.556 (0.454–0.658)	0.727 (0.539–0.879)	0.385 (0.208–0.538)	1.182 (0.876–1.595)	0.709 (0.584–0.857)	0.429 (0.255–0.608)	0.690 (0.549–0.813)
Model 2	0.754 (0.652–0.857)	0.909 (0.606–1.000)	0.519 (0.288–0.654)	1.891 (1.398–2.559)	0.175 (0.000–0.602)	0.545 (0.364–0.719)	0.900 (0.790–0.968)
Model 3	0.734 (0.624–0.843)	0.606 (0.333–0.788)	0.808 (0.481–0.923)	3.152 (1.693–5.866)	0.488 (0.441–0.722)	0.667 (0.482–0.820)	0.764 (0.632–0.875)
Model 4	0.759 (0.657–0.861)	0.848 (0.545–0.970)	0.615 (0.308–0.750)	2.206 (1.520–3.203)	0.246 (0.098–0.606)	0.583 (0.392–0.745)	0.865 (0.742–0.944)
Model 5	0.831 (0.741–0.921)	0.788 (0.485–0.909)	0.827 (0.385–0.923)	4.552 (2.449–8.462)	0.257 (0.236–0.558)	0.743 (0.577–0.889)	0.860 (0.742–0.944)

AUC: area under ROC curve, CI: confidence interval, SEN: sensitivity, SPE: specificity, PLR: positive likelihood ratio, NLR: negative likelihood ratio, PPV: positive predictive value, NPV: negative predictive value.

## Data Availability

The datasets used and/or analysed during the current study are available from the corresponding author on reasonable request.

## Supplementary Materials

The following supporting information can be downloaded at: https://www.mdpi.com/article/10.3390/diagnostics16010118/s1. Figure S1. Representative examples of coronary CTA analysis. Figure S2. Principal component analysis of coronary CTA parameters (A) and cervical arterial CTA parameters (B). Figure S3. Principal component analysis of pericoronary adipose tissue radiomic features (A) and cervical perivascular cervical adipose tissue radiomic features (B). Figure S4. Intra-observer and inter-observer agreement analysis of measured values. Figure S5. Characteristics of the distribution of right CCA (A,B) and LM + LAD fibro-lipid (C,D) in the cerebral ischaemia event versus non-event groups. Figure S6. Lasso regression screening of PCAT radiomics features. Figure S7. Lasso regression screening of cervical arteries radiomics features. Figure S8. Relative importance of radiomics features. Figure S9. Forest plots of multivariate logistic regression analyses for Radscore_coronary_ and Radscore_cervical_. Figure S10. ROC curves of perivascular adipose tissue predicting the occurrence of IS/TIA in CAD patients. Figure S11. Incremental Value of Radiomic Features for Risk Stratification of Cerebral Ischaemic Events (Validation Cohort). Table S1: Clinical baseline characteristics of patients in the training and validation sets. Table S2: Clinical baseline characteristics of patients with or without cerebral ischaemic events. Table S3: Characterization of imaging parameters of cervical CTA and coronary CTA (validation set). Table S4: Supplementary details of cervical artery CTA imaging parameter characteristics (training set). Table S5: Characterisation of high-risk plaques in cervical artery and coronary lesion locations. Table S6: Results of univariate and multivariate logistic regression analyses of clinical characteristics. Table S7: Results of univariate and multivariate logistic regression analyses of coronary CTA parameters. Table S8: Results of univariate and multivariate logistic regression analyses of cervical arterial CTA parameters. Table S9. Diagnostic performance metrics for different models in the training set.

## Abbreviations

The following abbreviations are used in this manuscript:

CCA	common carotid artery
FAI	fat attenuation index
HRP	high-risk plaque
IS	ischaemic strokes
LAD	left anterior descending artery
LCX	left circumflex artery
LM	left main
ICA	internal carotid artery
PCAT	pericoronary adipose tissue
PVAT	perivascular adipose tissue
PFD	perivascular fat density
RCA	right coronary artery
TIA	transient ischaemic attacks
VA	vertebral artery
