# Supplementary material for: Improving Risk Stratification for Transient Ischaemic Attacks and Ischaemic Stroke in Patients with Coronary Artery Disease: A Combined Radiomics Analysis of Multimodal Adipose Tissue
Source: Diagnostics (Basel). 2026 Jan 1;16(1):118. doi: 10.3390/diagnostics16010118 (PMC12785820; doi:10.3390/diagnostics16010118)
Supplement: Supplementary file 1 [file diagnostics-16-00118-s001.zip › diagnostics-3984390-supplementary.pdf]

Supplementary Materials

Definition of clinical parameters

Relevant indicators were defined as follows, hyperlipidaemia was defined as the presence of one of the following conditions, fasting total cholesterol > 6.2 mmol/L, low-density lipoprotein (LDL) cholesterol > 3.4 mmol/L, high-density lipoprotein (HDL) cholesterol < 1.0 mmol/L, triglyceride > 1.7 mmol/L, or a diagnostic record of dyslipidaemia; hypertension: systolic blood pressure > 140 mmHg and/or diastolic blood pressure > 90 mmHg; diabetes mellitus was defined as the presence of a history of diabetes mellitus or a fasting blood glucose ⩾ 7.0 mmol/L; the triglyceride-glucose index (TyG) was calculated by the formula: ln (fasting blood glucose * fasting triglyceride)/2.

CTA scanning programme

At Institution 1, coronary CTA was performed using either a SOMATOM Force CT scanner (Siemens Healthineers, Germany) or an Aquilion ONE 320-slice CT scanner (Canon Medical Systems, Japan). A prospective electrocardiogram (ECG)-gated protocol was employed with the following parameters: tube voltage, 120 kV; tube current, 300 – 500 mA (adjusted according to body mass index); and temporal resolution, 275 ms. Scanning commenced 6 s after contrast agent injection. Under regular cardiac rhythm, 65 mL of iodinated contrast medium (Qminipaque, 350 mg/mL; GE Healthcare, USA) was administered at a flow rate of 4.5 mL/s, followed by a 20 mL saline flush at the same rate. Images were reconstructed with a 512 × 512 matrix and a slice thickness of 0.75 mm.

At Institution 2, coronary CTA was performed on a Brilliance 256 iCT scanner (Philips Healthcare, Netherlands) using a prospective ECG-gated protocol. Scanning parameters included a tube voltage of 120 kV, tube current of 300 – 500 mA (adjusted by body mass index), and a temporal resolution of 275 ms. Image acquisition began 6 s after contrast agent injection. For patients with regular cardiac rhythm, 60 mL of iodinated contrast medium (XENETIX®, Iodixanol Injection, 350 mg/mL; GUERBET, France) was injected at 4 mL/s over 15 s, followed by a 20 mL saline flush at 4 mL/s. Reconstructed images had a 512 × 512 matrix and a slice thickness of 0.9 mm.

Cervical artery CTA examinations were performed using the same CT scanners described above. Scans were acquired in spiral mode, covering the region from the aortic arch to the level of C1. At Institution 1, cervical artery CTA was performed with the following parameters: tube voltage, 120 kV; tube current, 300 – 500 mA (adjusted according to body mass index). A total of 75 mL of iodinated contrast medium (Qminipaque, 350 mg/mL; GE Healthcare, USA) was injected at a rate of 4 mL/s, followed by a 20 mL saline flush at the same rate via power injector. The detection plane was positioned at the level of the tracheal bifurcation, with the region of interest placed on the descending aorta. Image acquisition was triggered using bolus tracking once the attenuation threshold of 160 HU was reached. Scanning parameters included a pitch of 1.0, rotation time of 350 ms, reconstruction slice thickness of 0.5 mm, and reconstruction interval of 0.5 mm.

At Institution 2, cervical artery CTA was performed with a tube voltage of 120 kV and tube current determined by body mass index. A total of 60 – 80 mL of iodinated contrast agent (XENETIX®, 350 mg/mL; GUERBET, France) was administered at a rate of 4 mL/s, followed by a 30 mL saline flush at the same rate via power injector. Acquisition was triggered using bolus tracking 5 seconds after the

aortic arch reached an attenuation threshold of 100 HU. Scanning parameters included a pitch of 1.0, rotation time of 350 ms, reconstruction slice thickness of 0.9 mm, and reconstruction interval of 0.5 mm.

Two experienced cardiovascular imaging researchers interpreted the imaging results obtained.

Additional description of CTA measurement parameters

For cervical arteries CTA analysis, high-risk plaque features included soft plaques, plaque ulceration, plaque neovascularisation, and plaque thickness ⩾3 mm, soft plaques are low-attenuation plaques with HU ranging from approximately 16-90 HU (median approximately 40-50 HU), which reflect intraplaque haemorrhage, lipid-rich necrotic core and a combination of fibrous components. Ulcerated plaques showed contrast extension beyond the plaque vascular lumen by at least 1 mm, whereas plaque neovascularisation was demonstrated by plaque enhancement after angiography. Plaque thickness was measured linearly over the largest cross-section of the plaque perpendicular to the long axis of the vessel.

Radiomics feature extraction

In previous studies, the RCA PCAT has been demonstrated to serve as an effective surrogate marker for total cardiac adipose tissue. Building on this evidence, the present study utilized the Research Portal V1.6 platform (United Imaging Intelligence) to extract radiomics features from RCA PCAT. Within this platform, an automated segmentation model based on the U-Net deep learning architecture was applied to delineate mask regions of cardiac and pericoronary adipose tissue. Image preprocessing included normalization, resampling (1×1× mm^3), and grey-value standardization (binWidth = 25) to satisfy radiomics phenotyping requirements. Feature extraction was performed using the standardised feature set provided by the PyRadiomics open-source library. Following preprocessing, a total of 2,264 radiomic features were extracted from RCA PCAT. These included 450 first-order features describing tissue Hounsfield Unit (HU) distribution, 14 shape features, 525 grey-level co-occurrence matrix (GLCM) features, 402 grey-level size zone matrix (GLSZM) features, 400 grey-level run length matrix (GLRLM) features, 125 neighbouring grey-tone difference matrix (NGTDM) features, and 348 grey-level dependence matrix (GLDM) features.

Following the delineation of the perivascular adipose tissue (PVAT) region of interest (ROI) around the cervical arteries in 3D Slicer, the raw medical images and corresponding ROI masks were exported separately in .nrrd format to ensure consistency between images and masks. Radiomics analysis was conducted using Jupyter Notebook in a Python 3.8 environment. Image preprocessing was performed using an initialized feature extractor, which included grey-value standardization (binWidth = 25), resampling to an isotropic resolution of 1 × 1 × 1 mm^3, and multi-scale Gaussian filtering (σ = 1.0, 2.0, and 3.0 mm) to capture texture features across multiple scales. Feature extraction was implemented primarily using the PyRadiomics (v3.0.1) and SimpleITK (v2.1.1) libraries. A total of 1,595 radiomic features were extracted from the Pericervical adipose tissue, comprising 14 shape features, 306 first-order features, 408 grey-level co-occurrence matrix (GLCM) features, 238 grey-level dependence matrix (GLDM) features, 272 grey-level run length matrix (GLRLM) features, 272 grey-level size zone matrix (GLSZM) features, and 85 neighboring grey-tone difference matrix (NGTDM) features. All extracted features were standardized using Z-score normalization.

Radiomics signature screening

Radiomics feature screening process, (1) (1) Preprocessing: Features underwent centring and standardisation. The Yeo-Johnson transformation was applied to improve feature distribution and stabilise variance. Near-zero variance filtering eliminated redundant features with negligible information content. (2) Highly correlated features (correlation coefficient >0.9) were removed by Spearman's correlation coefficient. (3) LASSO regression using 10-fold cross-validation. (4) Plot lambda path diagram and Lasso regression cross-validation. (5) Extract non-zero coefficients to calculate Radscore (using lambda.min as the optimal lambda value).

Statistical analyses

Continuous variables following a normal distribution are presented as mean (SD), whereas non-normally distributed variables are expressed as median [interquartile range]. Categorical variables are reported as n (%). Between-group comparisons were performed using the *t*-test, Mann–Whitney *U* test, and chi-square test, as appropriate. Intra- and inter-observer agreement for categorical variables was assessed using Cohen’s kappa statistic, while agreement for continuous variables was evaluated using intraclass correlation coefficients (ICCs) and visualised with Bland–Altman plots.

Radiomics calculation formula

The $Radscore_{coronary}$ was derived from the weighted coefficients of eight pericoronary adipose tissue radiomic features, while the $Radscore_{cervical}$ was calculated based on the weighted coefficients of sixteen pericervical adipose tissue radiomic features.

$Radscore_{coronary}$=0.0548751774554633*laplaciansharpening_firstorder_10Percentile+0.0478902632908008*laplaciansharpening_firstorder_RootMeanSquared+0.0316031033977954*wavelet_firstorder_wavelet.LLL.Minimum+-0.0210216102785453*shotnoise_glszm_SmallAreaHighGrayLevelEmphasis+-0.0211079541324983*specklenoise_glszm_ZoneEntropy+-0.028919985512837*wavelet_ngtdm_wavelet.LLL.Contrast+-0.037114242482668*boxmean_ngtdm_Contrast+-0.0680084366657823*discretegaussian_firstorder_InterquartileRange+-0.288265562586073

$Radscore_{cervical}$=0.159336736208036*wavelet-HHL_firstorder_Skewness+0.14760254331114*original_shape_Sphericity+0.115326567260608*wavelet-HHL_gldm_DependenceVariance+0.0895357851304523*wavelet-LLH_firstorder_Median+0.0694833885646576*log-sigma-3-mm-3D_gldm_LowGrayLevelEmphasis+0.0522152989237808*log-sigma-1-mm-3D_glszm_SmallAreaLowGrayLevelEmphasis+0.00529426144365983*exponential_glszm_SizeZoneNonUniformityNormalized+-0.0174577329789579*log-sigma-3-mm-3D_firstorder_Maximum+-0.0209280981907256*logarithm_ngtdm_Contrast+-0.0446263845484799*wavelet-HLH_glcm_MCC+-0.064556410396096*wavelet-HHH_firstorder_Skewness+-0.0717385310283565*wavelet-HLL_glcm_ClusterShade+-0.101605903480579*wavelet-LLH_glcm_Correlation+-0.106043076961302*wavelet-HLH_glszm_SizeZoneNonUniformityNormalized+-0.11493382930595*exponential_glszm_ZoneVariance+-0.171966742522214*wavelet-HHH_glszm_SmallAreaEmphasis+-0.295928216770363

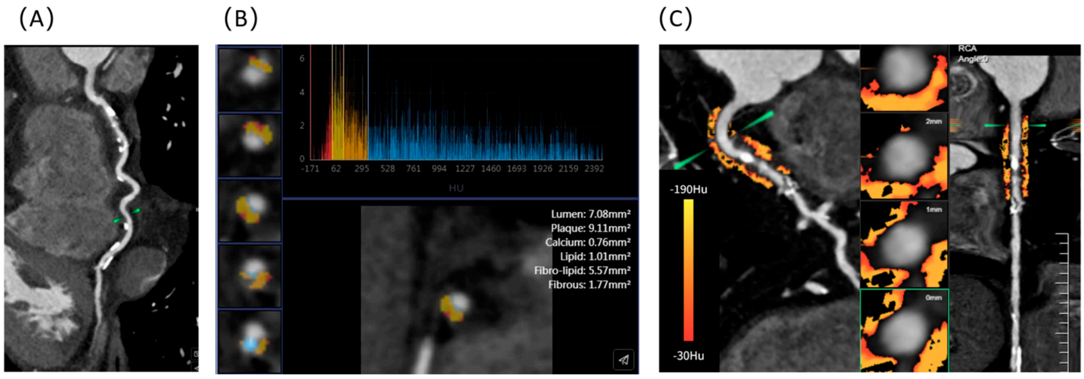

Figure S1. Representative examples of coronary CTA analysis. Shown are the imaging findings of a 65-year-old male patient with coronary artery disease: (A) Curved planar reformation of a coronary artery; (B) Plaque composition analysis, where red indicates lipid-rich necrotic core, yellow represents fibro-fatty plaque, orange denotes fibrous plaque, and blue corresponds to calcified plaque; (C) PCAT Measurement Map. PCAT represents a 3D ROI automatically identified across multiple consecutive slices. The figure illustrates the curved planar reformation and cross-sectional views, respectively. The colour-coded areas represent adipose tissue with attenuation values ranging from -190 to -30 HU. CTA: computed tomography angiography, PCAT: pericoronary adipose tissue.

Table S1：Clinical baseline characteristics of patients in the training and validation sets.

Patients	ALL(n=326)	Training set (n=241)	Validation set (n=85)	P
Age(years)	63.30 (9.17)	63.30 (9.46)	63.30 (8.36)	0.972
Male	202 (62.0%)	148 (61.4%)	54 (63.5%)	0.829
BMI(kg/m²)	24.80 (3.27)	24.90 (3.27)	24.70 (3.26)	0.598
Hypertension	214 (65.6%)	158 (65.6%)	56 (65.9%)	0.999
Diabetes mellitus	114 (35.0%)	82 (34.0%)	32 (37.6%)	0.638
Smoking	89 (27.3%)	64 (26.6%)	25 (29.4%)	0.714
Hyperlipidemia	186 (57.1%)	138 (57.3%)	48 (56.5%)	1.000
Heart rate(times/minute)	78.0 0[70.00;84.00]	78.00 [70.00;85.00]	76.00 [70.00;82.00]	0.292
Blood glucose (mmol/L)	6.37 (2.14)	6.35 (2.14)	6.43 (2.16)	0.769
Total cholesterol (mmol/L)	4.65 (1.22)	4.70 (1.23)	4.53 (1.20)	0.291
Triglycerides (mmol/L)	1.42 [1.05;1.98]	1.42 [1.10;1.97]	1.39 [0.99;1.99]	0.559
TyG Index	8.82 [8.45;9.25]	8.83 [8.47;9.18]	8.82 [8.41;9.30]	0.738
Pharmacotherapy (Hypertension)	144 (44.1%)	107 (44.4%)	37 (43.5%)	0.991
Cerebral ischaemic events	140 (42.9%)	107 (44.4%)	33 (38.8%)	0.444
Categories of cerebral ischaemic events				0.455
No cerebral ischaemic events	186 (57.1%)	134 (55.6%)	52 (61.2%)	
Ischaemic strokes	98 (30.1%)	77 (32.0%)	21 (24.7%)	
Transient ischaemic attacks	42 (12.9%)	30 (12.4%)	12 (14.1%)	

BMI = body mass index.

Table S2：Clinical baseline characteristics of patients with or without cerebral ischaemic events

Patients	ALL (n=326)	Cerebral ischaemic events (n=140)	No cerebral ischaemic events (n=186)	P
Age(years)	63.30 (9.17)	63.0 (8.83)	63.5 (9.44)	0.631
Male	202 (62.0%)	95 (67.9%)	107 (57.5%)	0.074
BMI (kg/m²)	24.80 (3.27)	25.0 (3.41)	24.7 (3.16)	0.477
Hypertension	214 (65.6%)	102 (72.9%)	112 (60.2%)	0.024
Diabetes mellitus	114 (35.0%)	56 (40.0%)	58 (31.2%)	0.125
Smoking	89 (27.3%)	37 (26.4%)	52 (28.0%)	0.856
Hyperlipidaemia	186 (57.1%)	82 (58.6%)	104 (55.9%)	0.714
Heart rate(times/minute)	78.0 0[70.00;84.00]	78.0 [70.0;84.0]	78.0 [70.0;84.8]	0.480
Blood glucose (mmol/L)	6.37 (2.14)	6.49 (1.97)	6.28 (2.27)	0.364
Total cholesterol (mmol/L)	4.65 (1.22)	4.54 (1.18)	4.74 (1.25)	0.146
Triglycerides (mmol/L)	1.42 [1.05;1.98]	1.69 (1.07)	1.64 (0.82)	0.693
TyG Index	8.82 [8.45;9.25]	8.85 [8.46;9.23]	8.81 [8.45;9.28]	0.681
Pharmacotherapy (Hypertension)	144 (44.1%)	69 (49.3%)	75 (40.3%)	0.133

BMI = body mass index.

(A)

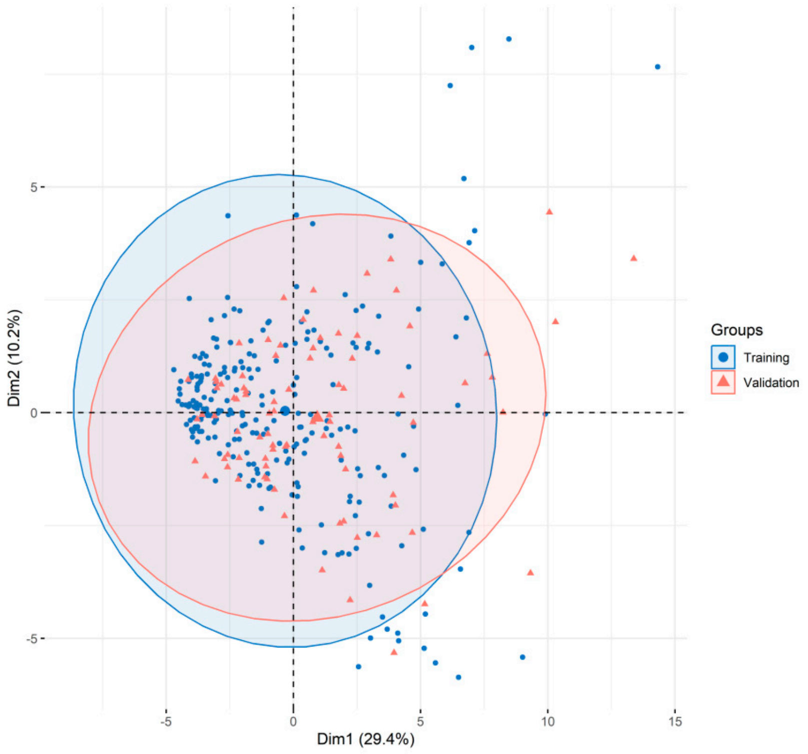

(B)

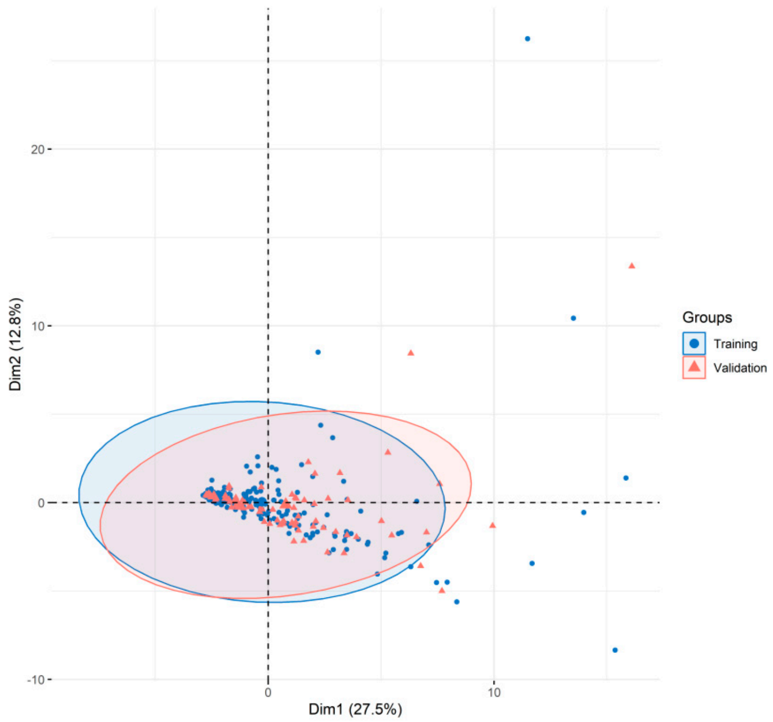

Figure S2. Principal component analysis of coronary CTA parameters (A) and cervical arterial CTA parameters (B).

Blue represents the training set data points, red represents the validation set data points, horizontal (Dim1) and vertical (Dim2) coordinates represent the first principal component (Principal Component 1, PC1) and the second principal component (Principal Component 2, PC2) after

dimensionality reduction, respectively, and the percentage in parentheses indicates the total variance explained by this principal component ratio.

(A)

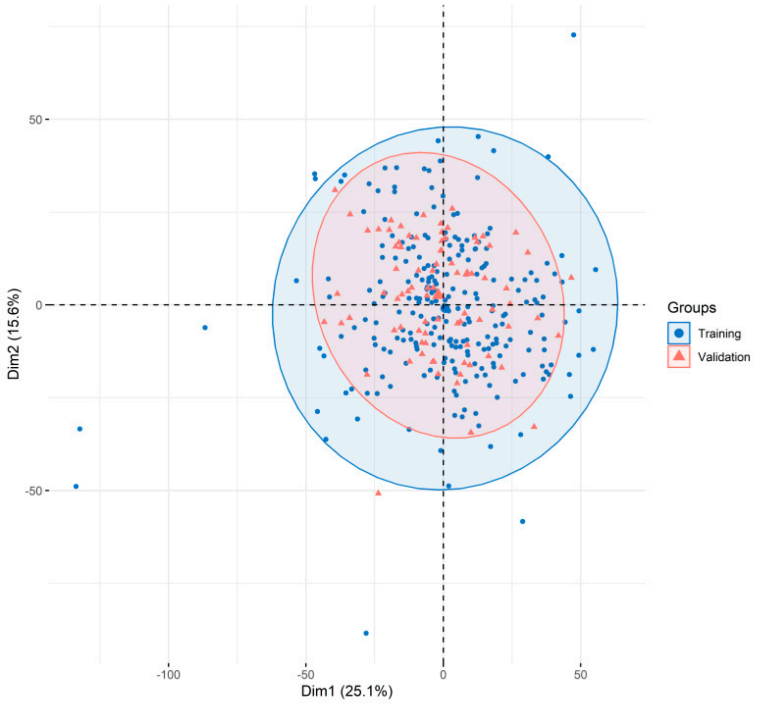

(B)

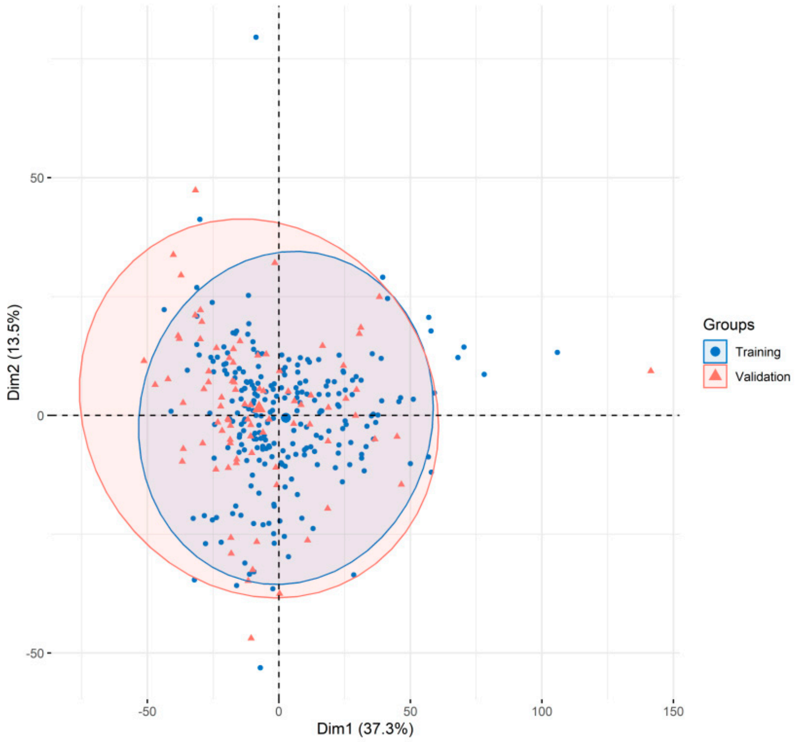

Figure S3. Principal component analysis of pericoronary adipose tissue radiomic features (A) and cervical perivascular cervical adipose tissue radiomic features (B).

Table S3：Characterization of imaging parameters of cervical CTA and coronary CTA（validation set）.

	Validation set (n=85)		
	No cerebral ischaemic events (n=52）	Cerebral ischaemic events(n=33)	p
LM+LAD total plaques	96.0 [32.1;178]	124 [40.1;186]	0.289
LM+LAD calcified plaque	25.2 [1.97;56.8]	40.9 [15.2;105]	0.096
LM+LAD non-calcified plaque	50.1 [14.5;113]	58.4 [27.5;101]	0.695
LM+LAD lipid	3.62 [0.77;13.2]	5.48 [1.73;9.69]	0.553
LM+LAD fibrous	17.3 [5.47;41.2]	28.7 [9.06;49.1]	0.232
LM+LAD fibro-lipid	20.1 [5.15;60.0]	16.4 [10.3;46.5]	0.860
LCX total plaques	6.40 [0.00;20.0]	1.96 [0.00;26.0]	0.888
LCX calcified plaque	0.00 [0.00;6.01]	0.00 [0.00;17.8]	0.893
LCX non-calcified plaque	1.80 [0.00;8.81]	1.14 [0.00;7.50]	0.438
LCX lipid	0.00 [0.00;0.57]	0.00 [0.00;0.00]	0.364
LCX fibrous	0.85 [0.00;5.34]	0.19 [0.00;2.40]	0.408
LCX fibro-lipid	0.00 [0.00;3.32]	0.00 [0.00;0.72]	0.712
RCA total plaques	59.4 [14.4;129]	59.1 [23.8;141]	0.684
RCA calcified plaque	10.9 [0.00;65.5]	16.6 [0.23;63.6]	0.689
RCA non-calcified plaque	32.2 [3.76;75.6]	35.2 [11.6;71.6]	0.461
RCA lipid	3.24 [0.00;7.79]	4.27 [0.50;7.65]	0.437
RCA fibrous	15.7 [3.07;31.5]	14.4 [6.50;30.9]	0.674
RCA fibro-lipid	12.6 [0.06;36.6]	17.6 [4.24;36.1]	0.393
LAD FAI	-79.55 (8.15)	-77.87 (8.00)	0.353
LCX FAI	-76.74 (8.31)	-73.67 (7.70)	0.086
RCA FAI	-84.05 (9.65)	-79.59 (9.99)	0.046
L.CCA calcified plaque	1.12 [0.00;71.5]	0.49 [0.00;28.2]	0.581
L.CCA non-calcified plaque	223 [0.00;534]	243 [1.18;445]	0.956
L.CCA fibrous	64.1 [0.00;144]	58.8 [0.97;136]	0.899
L.CCA fibro-lipid	115 [0.00;294]	110 [0.21;281]	0.949
L.CCA lipid	1.98 [0.00;44.6]	2.77 [0.00;26.3]	0.694

L.CCA total plaques	281 [0.00;618]	279 [1.18;548]	0.935
L.ICA calcified plaque	30.5 [6.50;87.2]	18.3 [3.49;113]	0.658
L.ICA non-calcified plaque	41.2 [7.30;350]	38.8 [9.43;371]	0.950
L.ICA fibrous	39.2 [7.30;117]	25.2 [8.74;124]	0.928
L.ICA fibro-lipid	0.29 [0.00;191]	6.18 [0.00;198]	0.786
L.ICA lipid	0.00 [0.00;4.34]	0.00 [0.00;2.84]	0.668
L.ICA total plaques	103 [15.6;579]	150 [14.8;473]	0.986
R.CCA calcified plaque	1.29 [0.00;46.6]	0.00 [0.00;7.51]	0.079
R.CCA non-calcified plaque	158 [0.00;457]	124 [0.00;313]	0.445
R.CCA fibrous	39.0 [0.00;142]	34.9 [0.00;75.5]	0.325
R.CCA fibro-lipid	87.5 [0.00;295]	86.4 [0.00;211]	0.507
R.CCA lipid	0.72 [0.00;28.8]	0.08 [0.00;6.10]	0.679
R.CCA total plaques	214 [0.00;595]	124 [0.00;341]	0.408
R.ICA calcified plaque	50.0 [4.08;123]	19.2 [6.56;117]	0.899
R.ICA non-calcified plaque	30.8 [7.66;277]	34.1 [6.84;518]	0.433
R.ICA fibrous	22.2 [6.16;127]	25.9 [6.70;151]	0.479
R.ICA fibro lipid	1.85 [0.00;94.9]	3.70 [0.05;218]	0.170
R.ICA lipid	0.00 [0.00;0.59]	0.00 [0.00;4.62]	0.290
R.ICA total plaques	94.1 [18.2;416]	102 [18.3;1007]	0.499
L.VA calcified plaque	0.00 [0.00;3.09]	0.00 [0.00;6.03]	0.493
L.VA non-calcified plaque	0.00 [0.00;13.1]	0.92 [0.00;14.9]	0.491
L.VA fibrous	0.00 [0.00;8.42]	0.00 [0.00;11.7]	0.600
L.VA fibro-lipid	0.00 [0.00;1.29]	0.29 [0.00;1.30]	0.532
L.VA lipid	0.00 [0.00;0.00]	0.00 [0.00;0.00]	0.835
L.VA total plaques	0.00 [0.00;24.4]	0.92 [0.00;23.2]	0.596
R.VA calcified plaque	0.00 [0.00;4.12]	0.00 [0.00;6.59]	0.771
R.VA non-calcified plaque	0.25 [0.00;12.9]	0.00 [0.00;11.8]	0.763
R.VA fibrous	0.25 [0.00;9.43]	0.00 [0.00;11.4]	0.710
R.VA fibro-lipid	0.00 [0.00;2.12]	0.00 [0.00;1.53]	0.582
R.VA lipid	0.00 [0.00;0.00]	0.00 [0.00;0.00]	0.396
R.VA.total plaques	0.32 [0.00;21.1]	0.00 [0.00;28.9]	0.756

PFD	-64.42 [-71.07;-58.01]	-58.26 [-67.52;-55.63]	0.076

LM: Left Main, LAD: left anterior descending artery, LCX: left circumflex artery, RCA: right coronary artery, FAI: fat attenuation index, R: right, L: left, CCA: common carotid artery, ICA: internal carotid artery, VA: vertebral artery, PFD: perivascular fat density.

Table S4: Supplementary details of cervical artery CTA imaging parameter characteristics (training set).

	Training set (n=241)		
	No cerebral ischaemic events (n=134)	Cerebral ischaemic events (n=107)	p
L.ICA lipid	0.00 [0.00;0.38]	0.00 [0.00;1.27]	0.349
R.CCA lipid	0.00 [0.00;4.97]	0.01 [0.00;9.66]	0.168
R.ICA lipid	0.00 [0.00;0.29]	0.00 [0.00;2.28]	0.193
L.VA calcified plaque	0.00 [0.00;2.13]	0.00 [0.00;1.27]	0.600
L.VA non-calcified plaque	0.00 [0.00;8.19]	0.00 [0.00;5.21]	0.460
L.VA fibrous	0.00 [0.00;6.50]	0.00 [0.00;4.54]	0.435
L.VA fibro-lipid	0.00 [0.00;0.53]	0.00 [0.00;0.13]	0.460
L.VA lipid	0.00 [0.00;0.00]	0.00 [0.00;0.00]	0.258
L.VA total plaques	0.00 [0.00;12.8]	0.00 [0.00;9.93]	0.478
R.VA calcified plaque	0.00 [0.00;0.13]	0.00 [0.00;0.14]	0.882
R.VA non-calcified plaque	0.00 [0.00;3.32]	0.00 [0.00;7.85]	0.539
R.VA fibrous	0.00 [0.00;2.66]	0.00 [0.00;4.18]	0.539
R.VA fibro-lipid	0.00 [0.00;0.10]	0.00 [0.00;0.96]	0.487
R.VA lipid	0.00 [0.00;0.00]	0.00 [0.00;0.00]	0.902
R.VA total plaques	0.00 [0.00;8.99]	0.00 [0.00;10.3]	0.616

Table S5: Characterisation of high-risk plaques in cervical artery and coronary lesion locations.

	Training set (n=241)			Validation set (n=85)		
	No cerebral ischaemic events (n=134)	Cerebral ischaemic events (n=107)	p	No cerebral ischaemic events (n=52）	Cerebral ischaemic events(n=33)	p
Analysis of coronary lesion sites						
Positive remodeling	72 (53.7%)	79 (73.8%)	0.002	29 (55.8%)	24 (72.7%)	0.179
Low attenuation plaque	54 (40.3%)	50 (46.7%)	0.384	23 (44.2%)	20 (60.6%)	0.212
Spotty calcification	43 (32.1%)	34 (31.8%)	1.000	6 (11.5%)	5 (15.2%)	0.743
Napkin ring sign	24 (17.9%)	12 (11.2%)	0.205	1 (1.92%)	2 (6.06%)	0.557
Coronary artery HRP	58 (43.3%)	56 (52.3%)	0.205	14 (26.9%)	19 (57.6%)	0.009
Coronary stenosis	54.0 [20.0;79.0]	56.0 [25.5;80.0]	0.091	57.5 [30.0;80.0]	65.0 [54.0;82.0]	0.221
MLA	4.42 [1.84;7.44]	3.77 [1.99;6.05]	0.228	3.47 [1.49;4.86]	3.32 [1.06;4.47]	0.629
Analysis of cervical vascular lesion sites						
Soft plaque	71 (53.0%)	60 (56.1%)	0.728	27 (51.9%)	21 (63.6%)	0.403
Plaque thickness	39 (29.1%)	37 (34.6%)	0.442	20 (38.5%)	10 (30.3%)	0.593
Plaque ulceration	4 (2.99%)	2 (1.87%)	0.696	1 (1.92%)	0 (0.00%)	1.000
Plaque enhancement	6 (4.48%)	7 (6.54%)	0.676	5 (9.62%)	3 (9.09%)	1.000
cervical artery HRP	71 (53.0%)	65 (60.7%)	0.282	27 (51.9%)	22 (66.7%)	0.265
cervical artery stenosis	25.0 [10.0;47.0]	23.0 [9.00;50.0]	0.868	42.5 [32.0;53.2]	52.0 [19.0;63.0]	0.346
MLA	15.0 [7.06;25.7]	16.9 [8.89;32.1]	0.209	11.4 [3.81;17.4]	7.28 [0.73;18.8]	0.611

HRP：high-risk plaque, MLA: Minimum Lumen Area, RI: Remodelling Index.

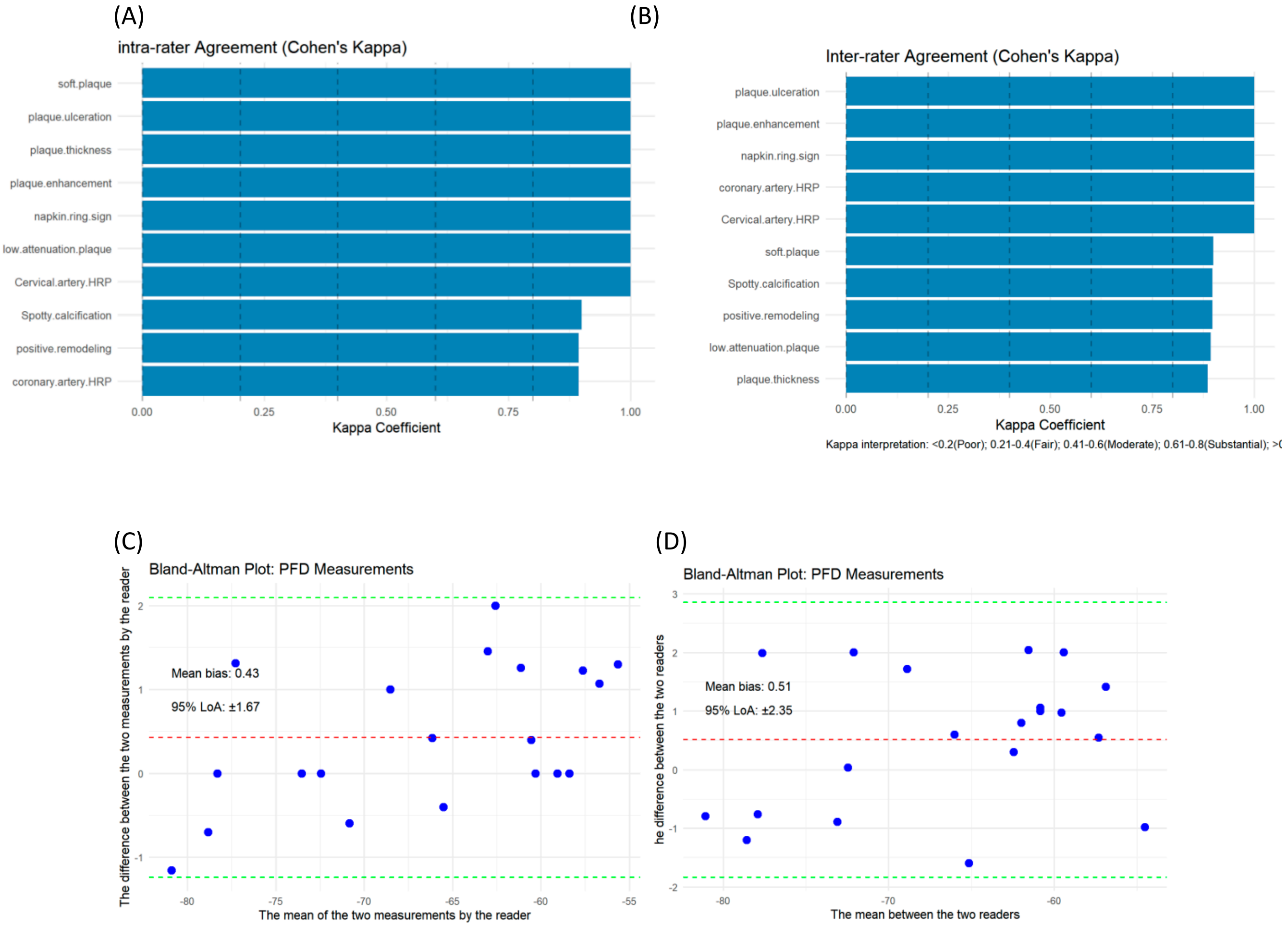

Figure S4. Intra-observer and inter-observer agreement analysis of measured values. Panels A, C present intra-rater agreement assessments for Reader 1 (measurements repeated at 6-month intervals). Panels B, D show inter-rater agreement between Reader 1 and Reader 2. Categorical variables (A, B): Analysed using Cohen's kappa statistic. Kappa interpretation: <0.2 (Poor); 0.21-0.4 (Fair); 0.41-0.6 (Moderate); 0.61-0.8(Substantial); >0.8 (Almost perfect). Continuous variables: Assessed via intraclass correlation coefficient (ICC) for inter-/intra-rater reliability. Intra-observer ICC = 0.993, inter-observer ICC = 0.988. Bland–Altman plots (illustrated for PFD, C, D): X-axis: Mean of paired measurements. Y-axis: Difference between measurements. Red dashed line: Mean bias. Green dashed line: 95% limits of agreement (LoA). PFD： pericarotid fat density.

Table S6: Results of univariate and multivariate logistic regression analyses of clinical characteristics.

	Univariable			Multivariable		
	**OR**	**CI**	**P**	**OR**	**CI**	**P**
Diabetes mellitus	1.41	0.82-2.41	0.209			
Age	0.99	0.97-1.02	0.695			
Male	1.57	0.92-2.66	0.095			
Hypertension	1.82	1.05-3.14	0.033	1.82	1.05-3.14	0.033
Smoking	0.81	0.45-1.45	0.479			
Drinking	1.20	0.56-2.56	0.632			
Heart rate	1.02	0.99-1.04	0.171			
BMI	1.02	0.94-1.10	0.679			
Blood glucose	1.08	0.96-1.21	0.226			
Total cholesterol	0.88	0.72-1.09	0.240			
Triglycerides	1.01	0.76-1.33	0.949			
Hyperlipidaemia	1.25	0.75-2.10	0.389			
TyG Index	1.11	0.72-1.72	0.639			

Abbreviations have same meanings as those in Table S1.

Table S7: Results of univariate and multivariate logistic regression analyses of coronary CTA parameters.

	Univariable			Multivariable		
	**OR**	**CI**	**P**	**OR**	**CI**	**P**
LM+LAD total plaques	1.001	0.999-1.003	0.364			
LM+LAD calcified plaque	1.000	0.997-1.003	0.824			
LM+LAD non-calcified plaque	1.006	1.001-1.011	0.031			
LM+LAD fibrous	1.011	0.998-1.024	0.089			
LM+LAD fibro-lipid	1.012	1.002-1.023	0.025	1.007	0.996-1.018	0.189
LM+LAD lipid	1.021	0.993-1.049	0.143			
LCX total plaques	0.998	0.993-1.004	0.522			
LCX calcified plaque	0.999	0.990-1.007	0.735			
LCX non-calcified plaque	0.996	0.987-1.006	0.451			
LCX fibrous	0.990	0.967-1.013	0.375			
LCX fibro-lipid	0.995	0.976-1.013	0.567			
LCX lipid	0.980	0.913-1.052	0.579			
RCA total plaques	1.000	0.999-1.002	0.667			
RCA calcified plaque	1.002	0.999-1.004	0.176			
RCA non-calcified plaque	1.001	0.998-1.004	0.615			
RCA fibrous	1.003	0.995-1.011	0.499			
RCA fibro-lipid	1.002	0.995-1.008	0.584			
RCA lipid	0.996	0.975-1.018	0.726			
Coronary stenosis	1.008	0.999-1.017	0.089			
LAD FAI	1.026	0.996-1.057	0.089			
LCX FAI	1.024	0.997-1.052	0.085			
RCA FAI	1.034	1.008-1.061	0.009	1.035	1.008-1.062	0.010
Analysis of high-risk plaques in coronary arteries						
Positive remodelling	2.430	1.403-4.206	0.002	2.359	1.329-4.189	0.003
Low attenuation plaque	1.300	0.778-2.171	0.317			
Spotty calcification	0.986	0.571-1.700	0.959			
Napkin ring sign	0.579	0.275-1.220	0.151			
Coronary artery HRP	1.439	0.863-2.397	0.163			
MLA	0.945	0.884-1.010	0.095			

LM+LAD non-calcified plaque excluded from multivariate factor analysis due to high covariance (VIF > 5). LM: Left Main, LAD: left anterior descending artery, LCX: left circumflex artery, RCA: right coronary artery, FAI: fat attenuation index, HRP：high-risk plaque, MLA: Minimum Lumen Area.

Table S8: Results of univariate and multivariate logistic regression analyses of cervical arterial CTA parameters.

	Univariable			Multivariable		
	**OR**	**CI**	**P**	**OR**	**CI**	**P**
L.CCA total plaque	1.000	1.000-1.001	0.250			
L.CCA calcified plaque	1.001	0.999-1.002	0.302			
L.CCA non-calcified plaque	1.000	1.000-1.001	0.349			
L.CCA fibrous	1.001	0.999-1.002	0.351			
L.CCA fibro-lipid	1.001	1.000-1.001	0.252			
L.CCA lipid	1.000	0.997-1.003	0.823			
L.ICA total plaque	1.000	1.000-1.001	0.122			
L.ICA calcified plaque	1.001	1.000-1.003	0.086			
L.ICA non-calcified plaque	1.000	1.000-1.001	0.308			
L.ICA fibrous	1.001	0.999-1.004	0.215			
L.ICA fibro-lipid	1.001	0.999-1.002	0.361			
L.ICA lipid	1.001	0.995-1.007	0.851			
R.CCA total plaque	1.000	1.000-1.001	0.080			
R.CCA calcified plaque	1.001	1.000-1.002	0.163			
R.CCA non-calcified plaque	1.000	1.000-1.001	0.116			
R.CCA fibrous	1.002	1.000-1.004	0.041	1.002	1.000-1.005	0.066
R.CCA fibro-lipid	1.001	1.000-1.002	0.257			
R.CCA lipid	1.002	0.998-1.005	0.269			
R.ICA total plaque	1.001	1.000-1.001	0.060			
R.ICA calcified plaque	1.001	1.000-1.003	0.065			
R.ICA non-calcified plaque	1.001	1.000-1.001	0.155			
R.ICA fibrous	1.001	0.999-1.004	0.250			
R.ICA fibro-lipid	1.001	1.000-1.002	0.169			
R.ICA lipid	1.004	0.997-1.011	0.302			
L.VA total plaque	0.998	0.996-1.001	0.193			
L.VA calcified plaque	0.996	0.987-1.005	0.345			
L.VA non-calcified plaque	0.998	0.995-1.001	0.205			
L.VA fibrous	0.993	0.983-1.002	0.142			
L.VA fibro-lipid	0.997	0.991-1.002	0.222			
L.VA lipid	0.991	0.974-1.009	0.340			
R.VA total plaque	1.001	0.999-1.002	0.400			
R.VA calcified plaque	1.002	0.998-1.007	0.323			
R.VA non-calcified plaque	1.001	0.999-1.003	0.466			
R.VA fibrous	1.004	0.997-1.010	0.300			
R.VA fibro-lipid	1.000	0.996-1.005	0.893			
R.VA lipid	1.000	0.991-1.010	0.949			
cervical artery stenosis	1.001	0.989-1.013	0.881			
PFD	1.035	1.007-1.063	0.013	1.032	1.005-1.061	0.021
Analysis of high-risk plaques in cervical artery						
Soft plaque	1.133	0.680-1.888	0.632			
Plaque thickness	1.288	0.746-2.222	0.364			

plaque ulceration	0.619	0.111-3.446	0.584
Plaque enhancement	1.493	0.487-4.583	0.483
cervical artery HRP	1.373	0.820-2.299	0.228
MLA	1.010	0.995-1.026	0.198

R: right, L: left, CCA: common carotid artery, ICA: internal carotid artery, VA: vertebral artery, PFD: perivascular fat density, HRP：high-risk plaque, MLA: Minimum Lumen Area.

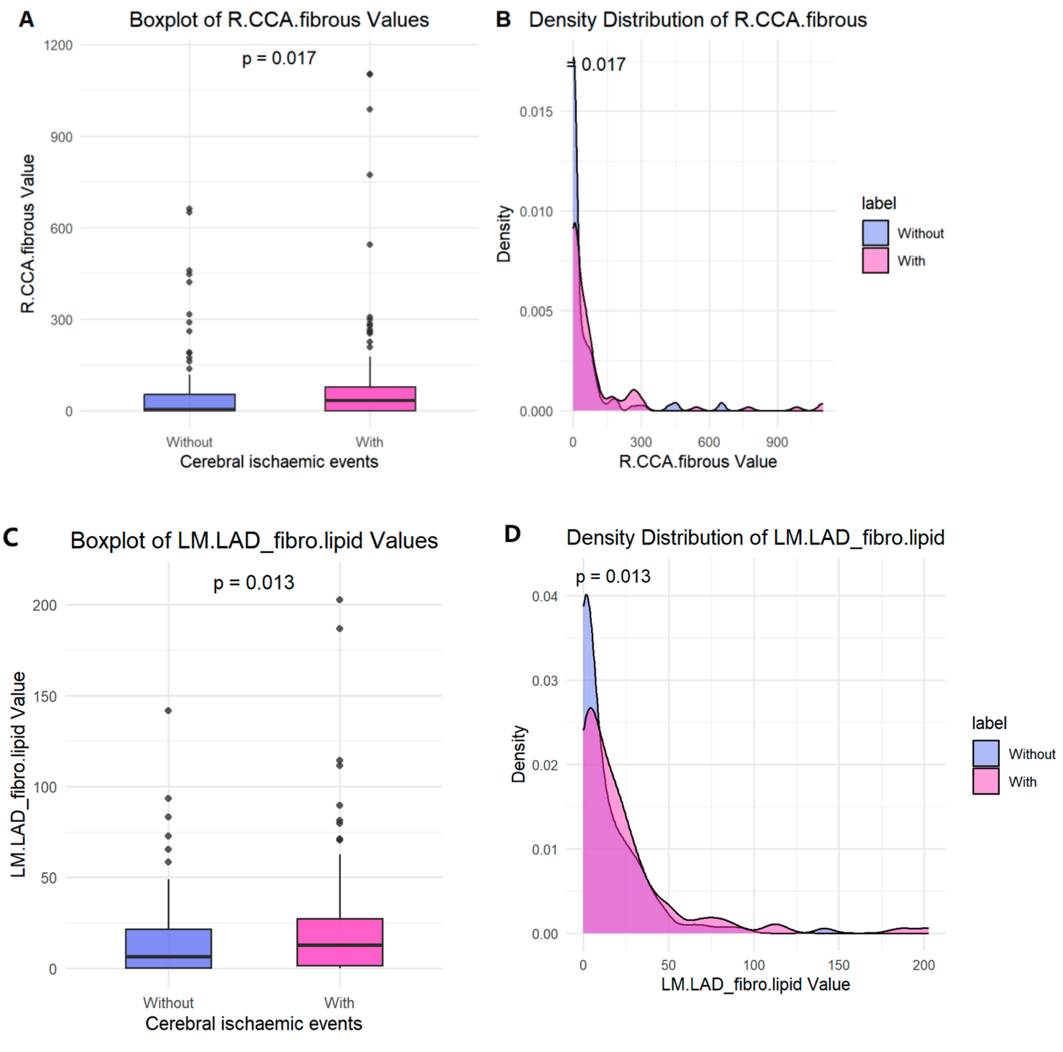

Figure S5. Characteristics of the distribution of right CCA (A-B) and LM+LAD fibro-lipid (C-D) in the cerebral ischaemia event versus non-event groups. left box-and-line plots demonstrate between-group differences, and right densitometric plots present the distribution of measured parameters. CCA: common carotid artery, LM: Left Main, LAD: left anterior descending artery.

(A) (B)

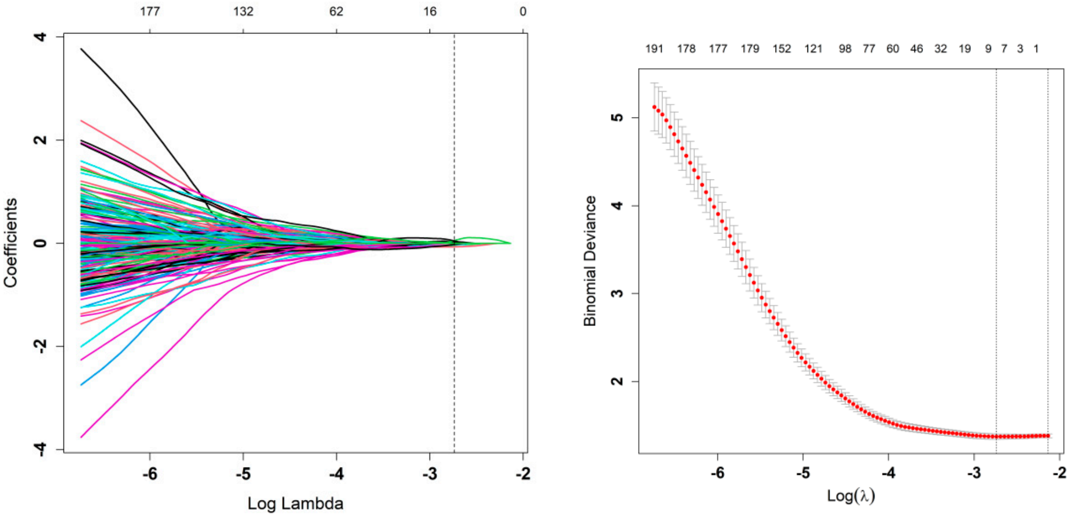

Figure S6. Lasso regression screening of PCAT radiomics features. (A) Lasso coefficient path diagram. (B) Lasso regression cross-validation plot (using 10-fold cross-validation). The dashed line on the left side corresponds to the minimum error, at which point lambda_min = 0.06463246, indicating that a total of 8 variables were included in the pericoronary adipose tissue radiomics score.

(A) (B)

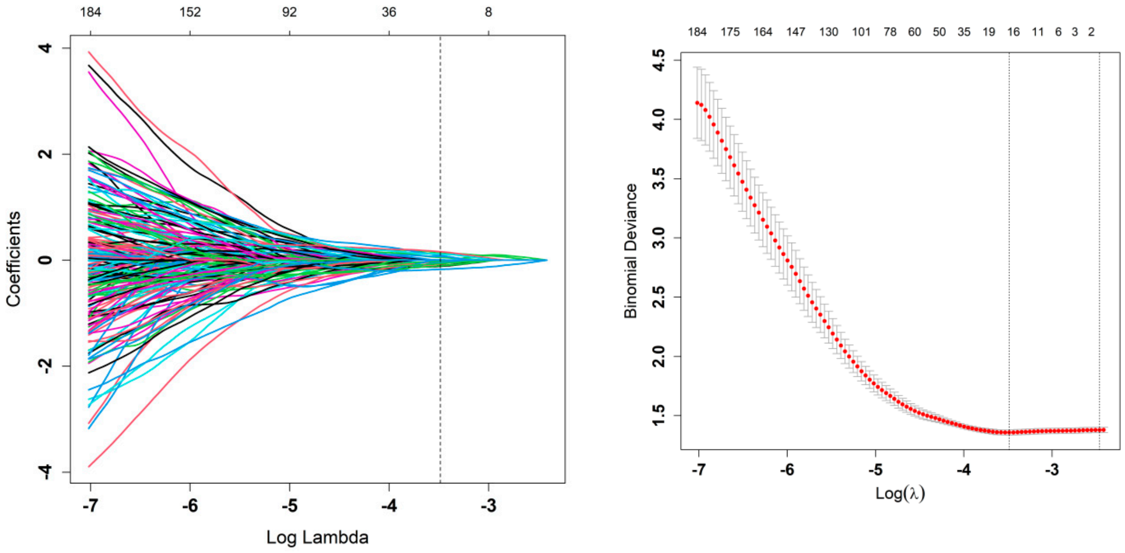

Figure S7. Lasso regression screening of cervical arteries radiomics features. (A) Lasso coefficient path diagram. (B) Lasso regression cross-validation plot (using 10-fold cross-validation). The dashed line on the left side corresponds to the minimum error, at which point lambda_min = 0.03068, indicating that a total of 16 variables were included in the pericervical adipose tissue radiomics score.

(A)

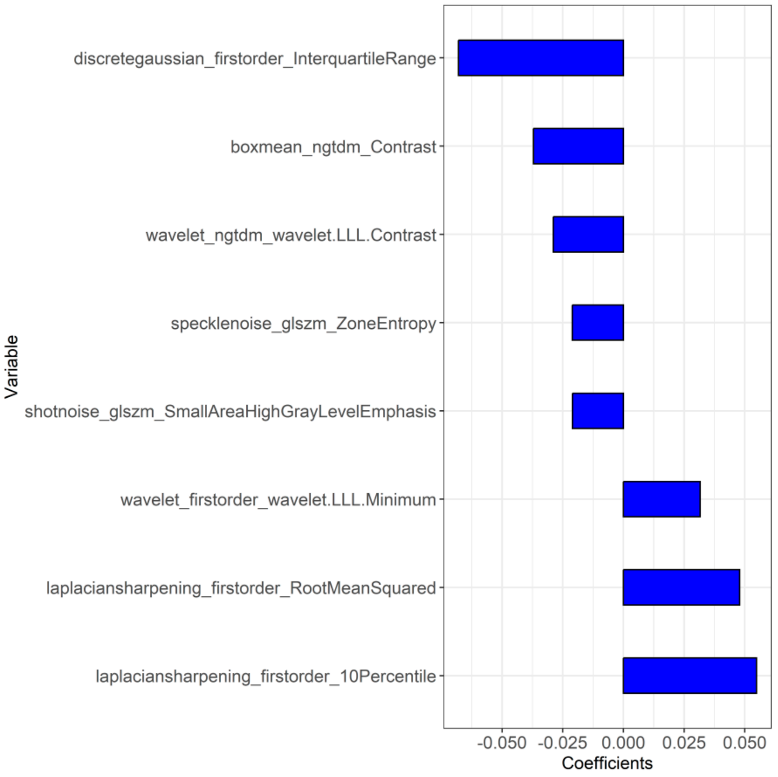

(B)

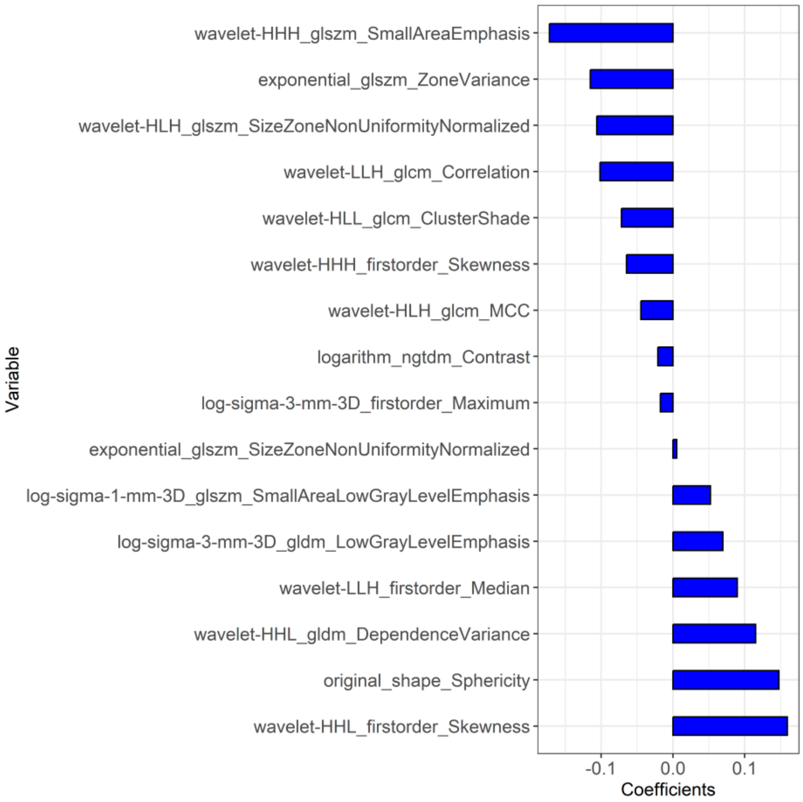

Figure S8 Relative importance of radiomics features. (A) $\text{Radscore}_{\text{coronary}}$ (B) $\text{Radscore}_{\text{cervical}}$. Horizontal coordinates are radiomics feature coefficients and vertical coordinates are names of radiomics features extracted by lasso regression analysis.

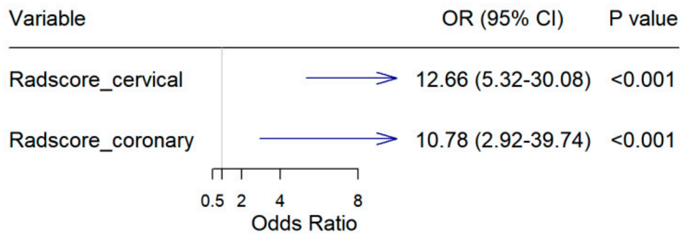

Figure S9. Forest plots of multivariate logistic regression analyses for $Radscore_{coronary}$ and $Radscore_{cervical}$.

Table S9. Diagnostic performance metrics for different models in the training set.

Model	Cutoff	AUC(95% CI)	SEN(95% CI)	SPE(95% CI)	PLR(95% CI)	NLR(95% CI)	PPV(95% CI)	NPV(95% CI)
Model 1	0.422	0.566 (0.507-0.625)	0.729 (0.605-0.819)	0.403 (0.280-0.500)	1.221 (1.019-1.463)	0.673 (0.645-0.790)	0.494 (0.397-0.594)	0.651 (0.562-0.730)
Model 2	0.402	0.698 (0.632-0.764)	0.738 (0.598-0.822)	0.582 (0.418-0.687)	1.767 (1.404-2.222)	0.450 (0.425-0.585)	0.585 (0.490-0.683)	0.736 (0.656-0.811)
Model 3	0.437	0.766 (0.704-0.827)	0.701 (0.542-0.794)	0.746 (0.560-0.828)	2.763 (2.015-3.788)	0.401 (0.367-0.553)	0.688 (0.595-0.777)	0.758 (0.680-0.831)
Model 4	0.523	0.711 (0.645-0.777)	0.579 (0.374-0.682)	0.791 (0.642-0.866)	2.773 (1.921-4.002)	0.532 (0.495-0.723)	0.689 (0.595-0.777)	0.702 (0.616-0.777)
Model 5	0.500	0.821 (0.769-0.873)	0.673 (0.533-0.766)	0.813 (0.657-0.888)	3.607 (2.473-5.260)	0.402 (0.356-0.526)	0.742 (0.644-0.819)	0.757 (0.672-0.824)

AUC: area under ROC curve, CI: confidence interval, SEN: Sensitivity, SPE: specificity, PLR: Positive Likelihood Ratio, NLR: negative likelihood ratio, PPV: positive predictive value, NPV: negative predictive value.

(A) (B)

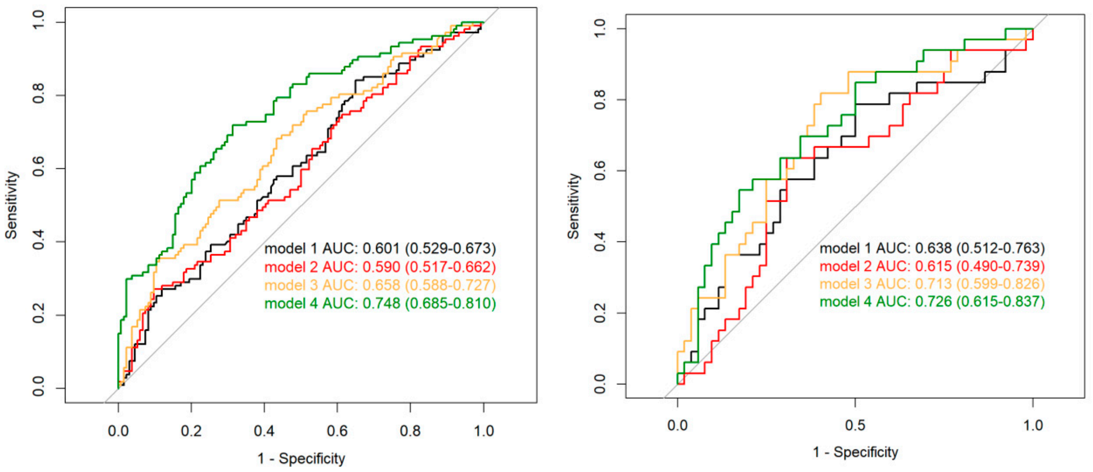

Figure S10. ROC curves of perivascular adipose tissue predicting the occurrence of IS/TIA in CAD patients.

Where model 1 is RCA FAI, model 2 is PFD, model 3 is Radscorecoronary, model 4 is Radscorecervical. (A) Training set, (B) Validation set. CAD: coronary artery disease, IS: ischaemic strokes, TIA: transient ischaemic attacks, RCA: right coronary artery, FAI: fat attenuation index, PFD: perivascular fat density.

(A) (B)

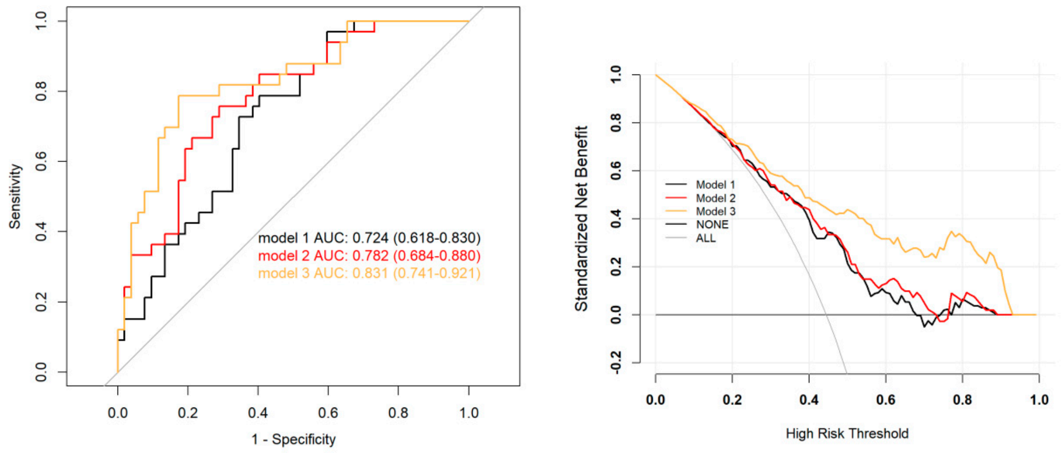

Figure S11. Incremental Value of Radiomic Features for Risk Stratification of Cerebral Ischaemic Events (Validation Cohort).

The incremental value of PVAT radiomic features was analysed in the validation cohort. In the figure: Model 1: Clinical + CTA imaging parameter model (including coronary CTA parameters and cervical CTA parameters); Model 2: Model 1 + $\text{Radscore}_{\text{coronary}}$; Model 3: Model 2 + $\text{Radscore}_{\text{cervical}}$. (A) ROC curves. (B) DCA curves. The results show that compared to the clinical+CTA feature model [AUC: 0.724 (0.618-0.830)], the stepwise addition of $\text{Radscore}_{\text{coronary}}$ and $\text{Radscore}_{\text{cervical}}$ to the model progressively increased the model's AUC value [Model 2 vs. Model 3 AUC: 0.782 (0.684-0.880) vs. 0.831 (0.741-0.921)].
